# GLP-1 Receptor Agonists and Tirzepatide in Men Seeking Fertility: A Structured Narrative Review and Proposed Clinical Framework

**DOI:** 10.3390/jcm15187313

**Published:** 2026-09-20

**Authors:** Aris Kaltsas, Vasileios Gkioxaris, Timoleon Giannakas, Athanasios Zachariou, Fotios Dimitriadis, Nikolaos Sofikitis, Michael Chrisofos

**Affiliations:** 1 Third Department of Urology, Attikon University Hospital, School of Medicine, National and Kapodistrian University of Athens, 12462 Athens, Greece; akaltsas@med.uoa.gr (A.K.); mchrysof@med.uoa.gr (M.C.); 2 Independent Researcher, 11526 Athens, Greece; gioxarisvasilis@gmail.com; 3 Department of Urology, Faculty of Medicine, School of Health Sciences, University of Ioannina, 45110 Ioannina, Greece; azachariou@uoi.gr (A.Z.); nsofikit@uoi.gr (N.S.); 4 Department of Urology, Faculty of Medicine, School of Health Sciences, Aristotle University of Thessaloniki, 54124 Thessaloniki, Greece; helabio@yahoo.gr

**Keywords:** GLP-1 receptor agonists, semaglutide, liraglutide, tirzepatide, male reproductive health, semen analysis, obesity, functional hypogonadism, paternal exposure, preconception counseling

## Abstract

Glucagon-like peptide-1 (GLP-1) receptor agonists and the dual glucose-dependent insulinotropic polypeptide (GIP)/GLP-1 receptor agonist tirzepatide are increasingly used to treat obesity and type 2 diabetes in men of reproductive age, but practical guidance for counseling men planning fatherhood remains limited. This structured narrative review distinguishes endocrine, semen, sexual-function, and clinical fertility outcomes. In metabolically impaired men, studies largely involving liraglutide or semaglutide report increases in total testosterone; free-testosterone findings are inconsistent, and the available small short-term studies did not show the marked gonadotropin suppression expected with exogenous testosterone. Some studies report favorable changes in semen parameters, but samples are small, populations heterogeneous, and medication effects cannot be separated reliably from weight loss. The present search identified no adequately powered GLP-1-specific prospective study that prespecified sperm DNA fragmentation, natural conception, assisted-reproduction outcomes, clinical pregnancy, or live birth. Sexual-function findings are mixed and do not establish causality. Evidence on paternal preconception exposure is insufficient, and pregnancy-related label precautions do not by themselves establish risk from paternal exposure. No eligible controlled tirzepatide-specific study assessing semen or clinical fertility outcomes was identified by this search. These therapies should be used for established metabolic indications, not as male infertility treatments. The proposed framework is author-developed, evidence-informed, non-validated, and intended for individualized counseling and prospective evaluation.

## 1. Introduction

Glucagon-like peptide-1 (GLP-1) receptor agonists—exenatide, liraglutide, dulaglutide, and semaglutide—and tirzepatide, a mechanistically distinct dual agonist of the glucose-dependent insulinotropic polypeptide (GIP) and GLP-1 receptors, are widely used for obesity and type 2 diabetes, produce clinically meaningful weight loss, and are increasingly prescribed to men of reproductive age [1,2,3,4]. Their andrological relevance reflects the association of obesity with functional hypogonadism, altered semen parameters, and sexual dysfunction, some of which may improve with weight reduction [1,5,6,7,8,9,10]. These observational associations do not establish that pharmacological weight loss improves fertility.

Biological plausibility derives from GLP-1 receptor expression in the testis and in human spermatozoa [11,12], although acute GLP-1 administration did not alter reproductive hormone secretion in healthy men [13]. The clinically decisive question is whether these drugs help the fertility-seeking male. Exogenous testosterone remains appropriate for selected men with confirmed symptomatic hypogonadism, but it suppresses gonadotropins and intratesticular testosterone and may impair spermatogenesis; it should not be used as monotherapy when current or future fertility is desired [14,15,16,17].

Existing reviews identify a more developed endocrine evidence base than a fertility evidence base, with overlapping primary studies and uncertain separation of drug effects from weight loss [1,5,18,19,20,21,22,23,24]. The present review focuses on integrating these findings with fertility intention, metabolic indication, and the couple’s reproductive timeline.

This structured narrative review separates metabolic and endocrine outcomes from semen and clinical fertility outcomes, considers classic GLP-1 receptor agonists separately from tirzepatide, and proposes an author-developed, evidence-informed, non-validated framework for individualized counseling. Its clinical focus is metabolic therapy in selected men with obesity or metabolic dysfunction who desire fertility [5,20].

## 2. Methods

### 2.1. Design and Scope

This is a clinically oriented narrative review of the literature on GLP-1 receptor agonists, the dual GIP/GLP-1 receptor agonist tirzepatide, and male reproductive health. It is not a systematic review, meta-analysis, or scoping review, and Preferred Reporting Items for Systematic Reviews and Meta-Analyses (PRISMA) reporting was not applied [25]; the work was structured a priori and guided by the Scale for the Assessment of Narrative Review Articles (SANRA) quality criteria [26]. The scope is the fertility-seeking male—an adult who desires current or future fertility. The category without current or anticipated fertility intention in the counseling framework (Section 10) is included as a triage boundary: it identifies men outside the fertility-seeking pathway, for whom routine reproductive testing is not proposed solely because treatment is considered. Fertility intention is reassessed if circumstances change. Endpoints are grouped a priori into hormonal endpoints (testosterone, sex hormone-binding globulin, luteinizing hormone, follicle-stimulating hormone, and estradiol), reproductive endpoints (semen parameters, sperm function, pregnancy, and live birth), and sexual function, so that the better-developed endocrine evidence is not conflated with the sparse fertility evidence [20]. Semen parameters are interpreted against the sixth-edition World Health Organization reference values [27,28], recognizing that the lower fifth-percentile limits describe a reference distribution and do not separate fertile from infertile men. Mechanistic findings are labeled by model type, and tirzepatide is treated separately as a dual GIP/GLP-1 receptor agonist [2].

### 2.2. Information Sources and Search

PubMed/MEDLINE was searched from database inception to 10 August 2026, the bibliographic eligibility cutoff. Reference lists of relevant reviews and included reports were checked iteratively to identify eligible reports published by that cutoff [1,5,20]. The search string was (“glucagon-like peptide 1”[tiab] OR “GLP-1”[tiab] OR semaglutide[tiab] OR liraglutide[tiab] OR dulaglutide[tiab] OR exenatide[tiab] OR tirzepatide[tiab] OR orforglipron[tiab] OR retatrutide[tiab]) AND (male[tiab] OR men[tiab] OR paternal[tiab] OR sperm*[tiab] OR semen[tiab] OR testosterone[tiab] OR hypogonadism[tiab] OR “erectile dysfunction”[tiab] OR fertilit*[tiab] OR preconception[tiab]). No other bibliographic database was searched, a limitation stated in Section 11.1. Supplementary Table S2 reports the platform, dates, complete queries, limits, and available audit information. Because the contemporaneous PubMed count was not retained, the unchanged query was rerun on 15 August 2026 to document its current yield. Directly relevant reviews first retrieved during this audit were appraised as contextual syntheses and used for citation chasing rather than to redefine the bibliographic eligibility cutoff. Citation chasing identified one full prospective cohort and one conference abstract published before the cutoff that met the review’s eligibility criteria and were incorporated into the evidence synthesis; reference-list checking was not logged as a separate record set. ClinicalTrials.gov was searched separately on 15 August 2026 using intervention, male-population, and reproductive-outcome terms, without restrictions by recruitment status, study type, phase, country, age, date, or language. Regulatory documents—United States Food and Drug Administration product labeling and safety communications—and guideline documents were retrieved directly from the issuing bodies. During revision, the intervention terms were expanded to include lixisenatide[tiab] and albiglutide[tiab]. On 10 September 2026, the original and expanded queries were run with identical publication-date limits through 10 August 2026. They retrieved 3150 and 3154 records, respectively. All four additional records were assessed for relevance; none were eligible for the male reproductive evidence synthesis. Complete queries, yields, and record-level exclusion reasons are documented in Supplementary Table S2. These current counts do not replace the historical audit yield or reconstruct the unavailable original search history.

### 2.3. Source Selection and Evidence Hierarchy

Eligible sources were human studies of any design, guideline and regulatory documents, and preclinical work used only to illustrate biological plausibility; publications in languages other than English were not assessed. Conference abstracts, case reports, and overlapping syntheses were eligible but were identified as such wherever they are cited and are treated as hypothesis-generating rather than as evidence of effect. Titles and abstracts were screened by A.K. and T.G.; full texts were assessed by both reviewers where obtainable. Sources available only as abstracts, or whose full reports could not be obtained, were appraised at abstract level and are explicitly identified as such. Study characteristics and numerical outcomes were extracted by T.G. and V.G. and checked by A.Z., F.D., and N.S. against the primary report where available. No registered protocol, duplicate independent screening, PRISMA flow diagram, or formal meta-analysis was undertaken.

### 2.4. Data Extraction and Critical Appraisal

Instrument-based risk-of-bias grading of individual studies, for example with the Risk Of Bias In Non-randomized Studies of Interventions (ROBINS-I) tool [29], was not undertaken. Consistent with the narrative design of this review, methodological quality was instead appraised descriptively and domain by domain, with attention to design, participant selection, comparator, outcome-specific sample size, attrition, confounding, concomitant interventions, outcome ascertainment, and duration of follow-up; this appraisal appears in the text. The absence of study-level risk-of-bias grading is stated among the limitations in Section 11.1. The Strengthening the Reporting of Observational Studies in Epidemiology (STROBE) statement is used only as a reporting standard against which the completeness of observational reports was judged, not as a risk-of-bias instrument [30]. Numerical values emphasized in the text and tables were checked against the cited primary reports when available; abstract-only evidence is explicitly identified as such wherever it is used.

For transparency, the tabulated inventory comprises 39 distinct reports: 13 primary human reports in the core evidence table and 26 additional reports in Supplementary Table S1. The core table includes five reports with semen endpoints and eight with endocrine endpoints only. Supplementary Table S1 contains 14 syntheses or reviews, five published clinical reports focused on sexual outcomes, two pharmacovigilance analyses, one genetic-proxy analysis, and four conference abstracts. These are report counts rather than independent study populations; outcome domains and underlying datasets may overlap. The mechanistic evidence comprises 14 experimental-model reports and two human native-GLP-1 infusion reports. Section 7 discusses eight clinical sexual-function reports, in addition to pharmacovigilance and genetic-proxy evidence. Two conference reports directly address paternal exposure in relation to pregnancy or perinatal outcomes. Domain-specific reference lists and counting rules are provided in Supplementary Table S2.

### 2.5. Framework Development

The initial framework was drafted by A.K. from the narrative evidence synthesis and the applicable guideline principles. The fertility-intention categories, assessment steps, and escalation and reassessment triggers were selected according to whether they could alter counseling, testing, referral, or treatment sequencing. Statements directly derived from existing guidelines were tagged [G], whereas pragmatic proposals generated by the present review were tagged [P]. All coauthors reviewed the draft iteratively for clinical relevance, methodological consistency, and concordance with the cited evidence, and the framework was revised in response to their comments. A.K. and T.G. subsequently translated the agreed structure into figures. No formal Delphi process, external stakeholder consultation, formal evidence-to-decision procedure, prospective evaluation, or external validation was undertaken. Agreement among the coauthors therefore represents internal author agreement rather than a formal consensus process. The framework should be regarded as a proposed, author-developed, evidence-informed, non-validated counseling aid for future evaluation, rather than as a guideline, a formally derived consensus statement, or a validated standard of care.

### 2.6. Figure Preparation and Use of Artificial Intelligence

The figures were prepared from author-written specifications using BioRender AI (BioRender: https://www.biorender.com, Toronto, ON, Canada), a web-based application with a generative artificial-intelligence layout feature; two of the figures were redrawn in the same application during revision to distinguish experimental models and to make the scope of the counseling framework explicit. A publication license is cited in each figure legend. Every element of each figure derives from the evidence synthesis reported here; the authors reviewed, corrected, and approved each version, and responsibility for source verification, evidence appraisal, clinical interpretation, and the final text and figures remains with the authors.

## 3. Pharmacological Background

Incretins—principally GLP-1 and GIP—are gut-derived peptides that potentiate glucose-dependent insulin secretion; beyond the islet, GLP-1 regulates appetite, gastric emptying, and body weight, and native GLP-1 has a short half-life that therapeutic agonists overcome through structural modification [31,32]. GLP-1 receptor agonists act selectively at the GLP-1 receptor. Exenatide was the first introduced, followed by liraglutide, dulaglutide, and semaglutide; dosing ranges from daily to once-weekly. They produce clinically meaningful weight loss—approximately 15% at 68 weeks with once-weekly semaglutide [3], which proved superior to daily liraglutide in a head-to-head randomized trial [33], with smaller reductions reported for liraglutide [34,35]—while glycemic control also improves [36]. Oral formulations and multi-receptor agonists are also being studied for metabolic indications [37,38,39]. OASIS 4 evaluated oral semaglutide 25 mg for weight management; its primary publication did not report semen, testosterone, or fertility outcomes [37].

Tirzepatide is mechanistically distinct and should not be described simply as a GLP-1 receptor agonist: it is a dual agonist of both the GIP and the GLP-1 receptors, and in the SURMOUNT-1 trial it reduced weight by approximately 15–21% [4]. Because the additional GIP component may confer different tissue effects, data from classic GLP-1 receptor agonists cannot be assumed to apply to it; its male-reproductive evidence comprises one non-randomized initiation study reporting short-term endocrine and body-composition outcomes [2] and one very small study of testosterone add-on in men who were already late responders to tirzepatide [40]. Neither evaluated semen parameters, sperm function, conception, pregnancy, or live birth, so tirzepatide should not be presented as having reproductive evidence equivalent to classic GLP-1 receptor agonists.

These agents are licensed for obesity and type 2 diabetes, not for any reproductive indication [1,5,22]. They are metabolic therapies. Any reproductive effect should currently be regarded as a possible downstream consequence of weight loss and metabolic improvement, or as an unproven direct effect, rather than as an established fertility indication.

## 4. Mechanistic Basis for Reproductive Effects

### 4.1. Biological Plausibility: GLP-1 Signaling in the Male Reproductive System

**Figure 1:**
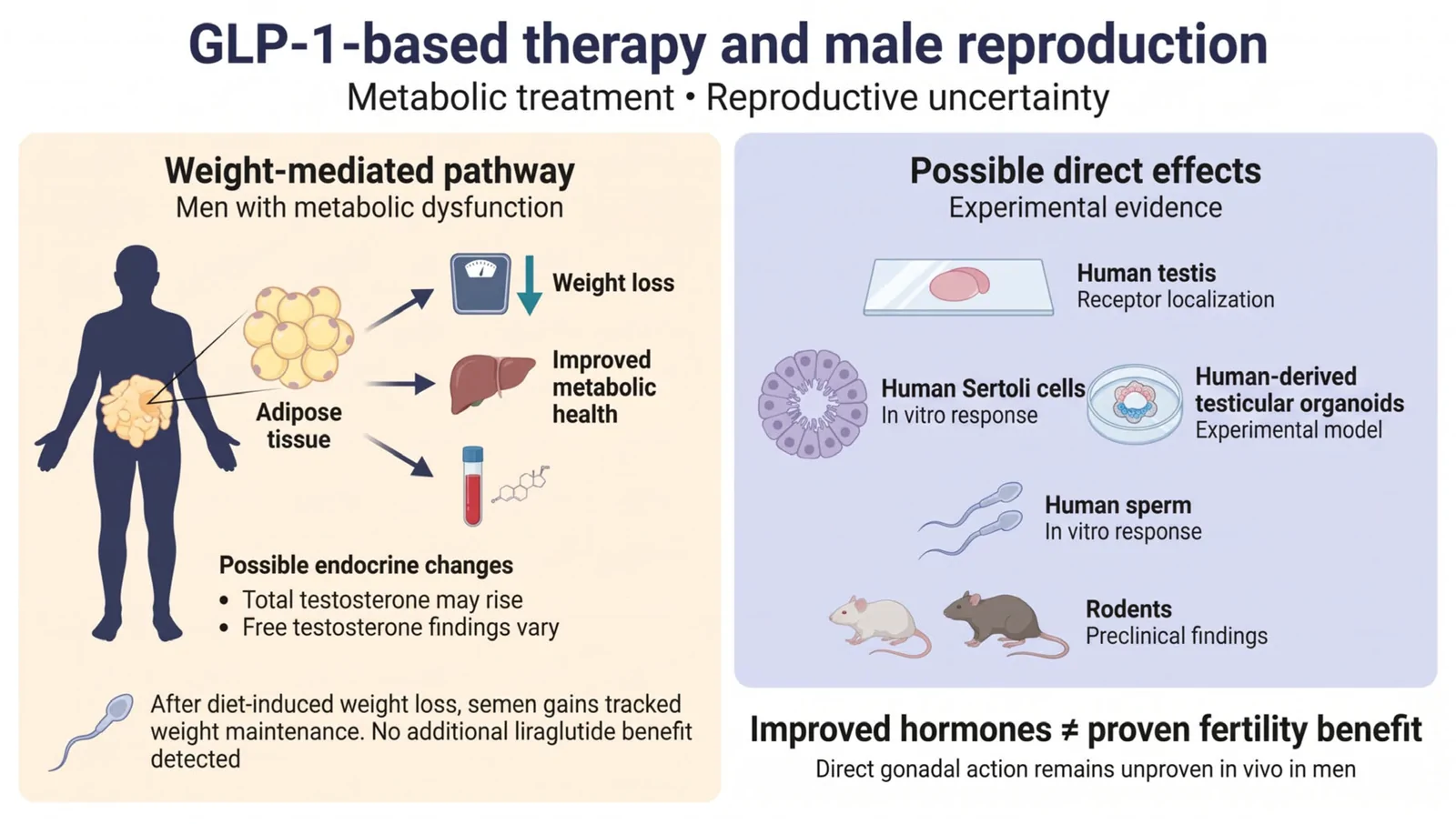
Weight-mediated pathways and experimental evidence for possible direct reproductive effects. The human clinical pathway is distinguished from receptor localization in tissue, functional responses in isolated human cells or sperm, human-derived organoids, and rodent models. Model-specific findings and limitations are detailed in Section 4 [11,12,13,41,42,43,44,45,46,47,48,49,50,51]. Sources for the weight-mediated pathway are cited in Section 4.2 and Section 5. Arrows indicate the proposed weight-mediated sequence, and ≠ denotes non-equivalence. An experimental response does not establish a direct in vivo human fertility effect. Abbreviations: GLP-1, glucagon-like peptide-1. Created in BioRender. Kaltsas, A. (2026) https://BioRender.com/94423ba.

The experimental evidence should be distinguished by what each model can establish: receptor detection or tissue localization, a functional response in vitro, and an effect in an intact organism are separate observations (Figure 1) [11,12,13,41,42,43,44,45].

In human testicular tissue, receptor localization to Leydig cells is an anatomical observation and does not by itself demonstrate receptor-mediated steroidogenesis or a response to treatment in vivo [12]. In cultured human Sertoli cells, GLP-1 altered metabolic and mitochondrial responses in a concentration-dependent manner; isolated-cell findings do not establish the behavior of the intact testis [44]. In ejaculated human spermatozoa, receptor detection was accompanied by an exendin-4-associated increase in progressive motility and changes in glucose-metabolizing enzyme activity and protein kinase A-dependent Akt activation in vitro; this provides functional experimental evidence but does not establish fertilization or clinical fertility benefit [11]. Human pluripotent stem cell-derived testicular organoids represent another experimental system: semaglutide reduced testosterone output and downregulated *INHBB*, but a developmental organoid does not reproduce all features of the adult testis or systemic drug exposure [45].

Animal and cell-line findings require separate interpretation. Liraglutide-associated mitochondrial improvement was reported in a mouse Leydig cell line without a detectable GLP-1 receptor, so that response cannot establish a receptor-mediated mechanism [41]. Rat Sertoli-cell and juvenile-rat studies examined cellular metabolism and blood–testis barrier permeability; the latter changes were reversible, with complete adult spermatogenesis and unchanged daily sperm production [42,43]. Rodent hypothalamic cell-line studies reported increased luteinizing hormone-releasing hormone secretion or increased *Kiss-1* expression [46,47]. In brain slices from ovariectomized female mice, liraglutide activated arcuate kisspeptin neurons, but treatment did not prevent fasting-associated luteinizing hormone suppression in vivo; this is indirect evidence for the male reproductive axis [48]. Exenatide reduced body weight and testicular inflammation while improving sperm motility and DNA integrity in mice, but weight-dependent and direct effects could not be separated [49]. Human infusion studies reported either no change in reproductive hormones or a reduction in pulsatile testosterone secretion [13,50]. A pair-fed diabetic rodent study provided an experimental comparison supporting weight-independent effects of tirzepatide within that model [51].

These systems support biological plausibility and identify testable mechanisms, but differ in species, developmental stage, exposure conditions, and measured outcomes. The present search identified no eligible in vivo human study establishing a direct gonadal effect that improves clinical fertility [5,11,12,13,44,45,50].

### 4.2. Weight-Mediated Versus Direct Gonadal Effects

GLP-1-based therapy may act through an indirect, weight-mediated pathway and a direct action on the gonad and reproductive axis (Figure 1); the weight-mediated pathway is better supported, and available human studies cannot cleanly separate the two [1,5].

In obesity, raised adipose aromatase activity, leptin, insulin resistance, and inflammation, together with low sex hormone-binding globulin, suppress hypothalamic gonadotropin-releasing hormone and produce a functional, largely reversible hypogonadotropic hypogonadism [52,53,54,55,56,57,58,59,60]. Weight loss by diet or bariatric surgery raises testosterone and reverses this state [61], and obesity-related oxidative stress and epigenetic change offer additional routes by which weight reduction might improve sperm [58,62,63,64,65,66,67]. In a randomized weight-maintenance substudy, an 8-week low-calorie diet increased sperm concentration and total sperm number; maintenance of those gains over 52 weeks tracked sustained weight loss irrespective of the randomized arm, and no additional semen benefit with liraglutide was detected; the substudy was not powered for between-arm reproductive comparison [68]. In the largest individual-level dataset, comprising 215 men with a mean weight loss of approximately 5%, total testosterone rose from 332 ± 134 to 399 ± 152 ng/dL and free testosterone from 6.9 ± 3.0 to 8.0 ± 4.2 ng/dL in the 61 men with paired measurements, the changes in total and free testosterone correlated modestly with the change in weight (*r* = −0.28 and −0.26, respectively), and the proportion with a normal testosterone concentration rose from 67% to 86%, consistent with but not demonstrating mediation by weight loss [69]. Bariatric surgery achieves larger endocrine gains still, with weighted mean differences in total testosterone of 5.46 to 8.02 nmol/L [70], although reproductive effects varied by procedure, so agent-specific data cannot be inferred from the bariatric literature [71]. The metabolic trials support the use of these agents for weight management; a sperm-specific action beyond weight loss was not established in the eligible clinical studies reviewed here [3,4,68].

A direct pathway remains plausible, but human mechanistic findings are discordant, and the present search identified no eligible in vivo human study confirming a direct gonadal action [11,12,13,23,44,46,47,48,50]. One meta-analysis restricted to GLP-1 receptor agonists and testicular dysfunction supports a predominantly weight-mediated interpretation [18], whereas a broader analysis of GLP-1 receptor agonists (8 studies, 375 men) and sodium–glucose cotransporter-2 inhibitors (3 studies, 52 men), assessed separately, reported a testosterone effect apparently independent of weight loss [19]. Because that inference rests on few small before–after studies and on aggregate data, and the largest individual-level dataset linked the testosterone response to the weight lost [69], it is best read as hypothesis-generating. This review therefore adopts a conservative hierarchy: the weight-mediated pathway is currently the best-supported explanation, whereas direct effects remain biologically plausible but unproven in vivo in men [18,22,43,45,49,72]. Their relative contributions cannot be quantified from the available designs [5,22,68].

## 5. Human Endocrine Outcomes

Across small, heterogeneous studies in metabolically impaired men, GLP-1 receptor agonist exposure was associated with higher total testosterone (Table 1). Because weight loss and rising sex hormone-binding globulin may increase total testosterone without a parallel increase in bioavailable androgen, these findings do not by themselves demonstrate greater testicular testosterone production. A systematic review of 10 studies (639 men) reported a consistent increase, particularly in men with obesity, type 2 diabetes, or functional hypogonadism, and two further reviews report the same direction of effect, although one also covers sodium–glucose cotransporter-2 inhibitors, analyzed separately [18,19,20]. Because these syntheses draw on an overlapping set of small primary studies—their counts differ (10 studies and 639 men in one, 7 studies pooled before–after and 680 men in another)—their agreement reflects shared source data rather than independent replication; the 10-study review was appraised at abstract level only [20]. In the before–after meta-analysis of seven studies, total testosterone rose with a standardized mean difference of 1.39 (95% confidence interval [CI] 0.70–2.09), but heterogeneity was extreme (*I*^2^ = 93%); removal of the study identified as its principal source—the non-randomized comparison in which allocation followed fertility intention [73]—lowered the estimate to 0.96 (95% CI 0.48–1.44) while leaving heterogeneity high (*I*^2^ = 83%). The effect correlated in meta-regression with the degree of weight and body-mass-index loss [18]. This parallels the established reversal of obesity-associated hypogonadotropic hypogonadism by weight loss and is therefore most plausibly weight-mediated [52,61]. An independent meta-analysis of four studies in 219 men reported a significant change in bioavailable testosterone alongside a fall in glycated hemoglobin, while free testosterone and sex hormone-binding globulin did not change significantly; numerical estimates from its abstract are not used because the reported signs and stated directions of effect are inconsistent [74].

Values are reported as they appear in the source: mean ± SD, median (interquartile range), or as printed where the source did not specify. Among these 13 reports, no comparative study selected participants specifically for active attempts at conception, and none was designed or powered for natural conception, clinical pregnancy, or live birth. Two additional 2026 reviews drew largely on the same primary studies and are treated as contextual syntheses rather than independent replication [83,84]. Aggregate descriptive counts from one are not used because its reported study and participant totals are inconsistent [83].

Conversely, in a six-month prospective cohort of 51 men with type 2 diabetes treated predominantly with extended-release exenatide, mean weight decreased by 2.27 kg and glycated hemoglobin by 0.7 percentage points, but total testosterone did not change significantly in the cohort overall. Exploratory subgroup analyses suggested a more favorable testosterone response among men with baseline total testosterone below 320 ng/dL or a reduction in glycated hemoglobin of at least one percentage point; no semen or clinical-fertility outcome was assessed [78]. An abstract-only retrospective review of 53 charts reported a mean total-testosterone increase of 111 ng/dL in the 14 men with paired measurements (*p* = 0.048), while the 2.4-point increase in the Sexual Health Inventory for Men (SHIM) score, available for only 5 men, was not significant (*p* = 0.42) and weight change was unrelated to testosterone change (*R*^2^ = 0.000, *p* = 0.969); the agents and treatment durations were not specified and no semen or clinical-fertility outcome was reported [85].

Two of the larger controlled primary studies pooled in those syntheses deserve individual mention, because each examined the relationship between weight change and testosterone. In a 12-week multicenter observational study of 176 men with obesity and type 2 diabetes, exenatide with metformin raised total testosterone by 121.72 ± 56.73 ng/dL against 34.67 ± 16.30 ng/dL with glimepiride and metformin (*p* < 0.001); within the exenatide arm the rise was 142.96 ± 41.55 ng/dL in the 65 men who lost at least 5% of body weight against 66.48 ± 54.12 ng/dL in the 25 who lost less (*p* < 0.001), and the change in total testosterone correlated inversely with the change in waist circumference (*r* = −0.443, *p* < 0.001), which alone explained 19.6% of its variance. Sex hormone-binding globulin rose from 18.54 ± 1.70 to 28.90 ± 1.50 nmol/L with exenatide and was unchanged with glimepiride, while neither free nor bioavailable testosterone differed between the arms after treatment (*p* = 0.884 and *p* = 0.840), so the larger rise in total testosterone was carried substantially by the binding protein rather than by an increase in bioavailable androgen [79]. In a 12-month retrospective study of 71 men with uncontrolled type 2 diabetes and mild-to-moderate erectile dysfunction, 30 of whom received dulaglutide or liraglutide added to metformin, total testosterone of at least 300 ng/dL was reached by 94% of those losing more than 10% of body weight against 3% of those losing less than 5% (*p* < 0.01), whereas attaining the glycated hemoglobin target was associated with a lower rather than a higher prevalence (15% against 51%, *p* < 0.01); sex hormone-binding globulin rose in every weight-loss stratum, and luteinizing hormone did not rise significantly in the men who lost most weight [82]. Neither study assessed semen parameters, sperm function, or any conception endpoint.

Free testosterone is less consistent, often offset by concurrent rises in sex hormone-binding globulin, which obesity lowers and weight loss restores [20,52]. Where sex hormone-binding globulin is altered, free testosterone should be measured by equilibrium dialysis or calculated from total testosterone, sex hormone-binding globulin, and albumin by a validated method rather than inferred from the total concentration [17]; few of the available studies meet this standard, and in the largest individual-level cohort paired free testosterone was available in only 61 of 215 men, although in that subset it rose in parallel with total testosterone [69]. In a 16-week randomized comparison in 30 obese men with functional hypogonadism, total testosterone rose with both liraglutide and transdermal testosterone, with weight loss in the liraglutide arm, and a prospective study reported increased testosterone and sex hormone-binding globulin with liraglutide [73,80]; estradiol is reported less consistently, although a reduction would be expected from decreased aromatization [52]. In a retrospective cohort, adding liraglutide to metformin and testosterone was accompanied by changes in testosterone and sex hormone-binding globulin, although the concurrent testosterone confounds attribution [81].

The gonadotropin response is the finding most often given clinical weight. Unlike exogenous testosterone, which suppresses luteinizing hormone and follicle-stimulating hormone, GLP-1 receptor agonists generally show no testosterone-like gonadotropin suppression in the small studies available, and some of those studies report increases—significantly so versus testosterone in the randomized comparison [20,80]. In the semaglutide trial, luteinizing and follicle-stimulating hormone were essentially unchanged [75]; for the dual GIP/GLP-1 receptor agonist tirzepatide, the standalone endocrine comparison identified by this review was the non-randomized initiation pilot [2]; a second small study evaluated testosterone undecanoate add-on in men already receiving tirzepatide and therefore cannot isolate the endocrine effect of tirzepatide itself, although in its tirzepatide-only arm total testosterone, calculated free testosterone, luteinizing hormone and follicle-stimulating hormone were all unchanged over six months in men who had lost less than 5% of body weight, a pattern compatible with a weight-related contribution but unable to distinguish mechanisms [40]. The appropriate reading of this is narrow. The available small studies did not show marked testosterone-like gonadotropin suppression; they do not establish preservation of the reproductive axis or spermatogenesis.

Consistent with the mechanistic hierarchy set out in Section 4.2, this endocrine pattern most likely reflects weight loss and metabolic recovery rather than a direct gonadotropic action: acute GLP-1 exposure did not increase gonadotropins in healthy men and has been reported to reduce the pulsatile component of testosterone secretion [13,50]. Two caveats apply throughout. The studies are small, short, and often open-label, with overlapping data across reviews, and their limited follow-up constrains inferences about durable testicular effects [20]. The findings also concern metabolically impaired men and should not be generalized to healthy eugonadal men, for whom the eligible studies reviewed here did not establish a semen benefit and hormonal data remain sparse [20,73,80].

## 6. Semen Parameters, Sperm Function, and Fertility Outcomes

In contrast to the endocrine signal, direct evidence on semen quality, sperm function, and clinical fertility is sparse and dominated by small studies, and the endpoints that matter most—pregnancy and live birth—are essentially absent [1,5,20,22]. The scale is worth stating plainly. Across the five small studies in Panel A of Table 1, approximately 93 men exposed to a GLP-1 receptor agonist had an on-treatment semen assessment, and only 61 analyzable participants came from randomized studies with a non-testosterone comparator. These counts are study- and outcome-specific, are restricted to the English-language reports summarized there, and do not represent evidence about clinical fertility.

### 6.1. Conventional Semen Parameters

For sperm concentration and count, the key evidence is a substudy of a randomized weight-maintenance trial in men with obesity in which an 8-week low-calorie diet—after which participants were randomized to placebo, exercise, liraglutide, or liraglutide plus exercise—increased sperm concentration approximately 1.5-fold and total sperm number approximately 1.4-fold; maintenance of these gains over 52 weeks was associated with sustained weight loss irrespective of the randomized arm [68]. The study did not establish a liraglutide-specific semen effect independent of weight-loss maintenance and was not powered for between-group reproductive comparisons. Semen volume and sperm motility (33.3% to 31.5%) did not change significantly, while the proportion of men with a very low motile sperm count (<1 million per ejaculate) fell from 6 of 47 to 1 of 47 (*p* < 0.05) [68]. Total motile sperm count, the parameter most closely tied to the route to conception, did not change significantly either after the diet phase (71.3 to 83.7 million per ejaculate, *p* = 0.1) or among weight-loss maintainers at 52 weeks (1.50-fold, 95% CI 0.64–3.52; *p* = 0.3) [68].

The clearest controlled semen data concern morphology. Twenty-five men with type 2 diabetes and functional hypogonadism were randomized to semaglutide or testosterone undecanoate for 24 weeks; serum testosterone rose in both arms, sperm concentration and total sperm number fell with testosterone, and with semaglutide, concentration, total sperm number, and motility did not change while morphologically normal forms rose from approximately 2% to 4% [75]. In this small open-label trial, semaglutide did not show the deterioration observed with testosterone, and morphology improved within the semaglutide arm. The actively spermatogenesis-suppressing comparator and absence of placebo or weight-matched control preclude a conclusion of fertility preservation or improvement [20,75]. Baseline semen quality in the cohort was poor: most parameters fell below the fifth percentile of the World Health Organization reference values for fertile men, with the exception of semen volume and sperm concentration, which lay between the fifth and tenth percentiles [75].

Motility has improved in some reports—with liraglutide in obese men of childbearing age, and with exendin-4 in vitro [11,73]—but the largest of the controlled semen studies is confounded by indication. In that prospective comparative study, allocation to liraglutide, gonadotropins, or transdermal testosterone followed fertility intention rather than randomization; the liraglutide arm consisted of 35 men explicitly without a current desire for fatherhood; a dietary intervention was applied in every group; and the reported improvements are principally within-group changes [73]. Allocation of this kind is a major confounder, because the arms differ systematically in the characteristic that also determines prognosis, so between-arm contrasts cannot be read as treatment effects, and the shared dietary co-intervention leaves any drug-specific contribution unidentifiable.

In healthy lean men, a randomized crossover trial of dulaglutide found no change in the only two sperm parameters it reported—concentration and progressive motility—a null result underpowered at 24 completers and four weeks of exposure, which is shorter than one spermatogenic cycle [76]. Systematic-review evidence points in the same overall direction—semen improvements in obese or hypogonadal but not healthy men—though certainty is low given few, small, heterogeneous studies [18,20]. One synthesis is an explicit exception and should not be read as concordant: in a meta-analysis of obesity interventions the pooled estimate for liraglutide was null and rested on two studies and 55 men (mean difference 0.58 million/mL, 95% CI from −24.76 to 25.91; *p* = 0.96; Grading of Recommendations Assessment, Development and Evaluation [GRADE] very low certainty); its authors concluded that the pharmacotherapy data were insufficient to support clear conclusions, and attributed the semen improvements they did observe to lifestyle intervention, apparently independent of the degree of weight lost [86].

### 6.2. Sperm-Function Outcomes

The present search identified no eligible clinical treatment study reporting sperm DNA fragmentation or a functional sperm endpoint beyond conventional semen parameters during GLP-1-based therapy. This clinical evidence gap should be distinguished from the in vitro human-sperm observations described in Section 4 [11]. Animal experiments, including a mouse study of dulaglutide, do not substitute for clinical endpoints [87]. In a meta-analysis of 12 weight-loss studies in 345 men with obesity, weight loss was associated with reduced DNA fragmentation index (effect size −0.689, 95% CI from −1.123 to −0.255; *p* = 0.002) and increased sperm concentration and progressive motility [88]. This provides a rationale for prospective measurement but cannot be attributed to GLP-1 receptor agonism. Selective clinical testing is addressed in Section 10.

### 6.3. Conception, Assisted Reproduction, Pregnancy, and Live Birth

The present search identified no eligible adequately powered GLP-1-specific prospective study evaluating natural conception, assisted-reproduction outcomes, clinical pregnancy, or live birth as prespecified endpoints, and the trials reporting semen endpoints did not follow couples to conception [20,68,86]. The nearest approach to a conception endpoint is a Danish nationwide registry cohort, available only as a 2026 conference abstract, in which male preconception semaglutide redemption was associated with higher odds of clinical pregnancy after intrauterine insemination (odds ratio 1.52, 95% CI 1.10–2.09) but lower odds across the first cycle of in vitro fertilization or intracytoplasmic sperm injection (0.75, 95% CI 0.58–0.96) [89]. Estimates that point in opposite directions across treatment modalities, are unadjusted for weight change, and have not undergone full peer review are hypothesis-generating and are not taken further here. At the opposite end of the evidence hierarchy, a single case abstract reported natural conception in a man with obesity, type 2 diabetes and azoospermia after sequential semaglutide and then tirzepatide, with total testosterone rising from 3.2 to 7.4 nmol/L and a subsequent sperm concentration of 31 million/mL [90]; its weight and body-mass-index values are internally inconsistent and are not used, sequential exposure to two agents cannot separate drug from weight loss, the azoospermic baseline was not reported as confirmed on a second specimen, and a single case is not evidence of efficacy. The evidence is also not uniformly favorable: a case report described worsening semen parameters during liraglutide exposure, with no spermatozoa on a repeat sample, followed by recovery of sperm concentration and motility to within reference limits five months after withdrawal; the fresh sample obtained at that visit was used for intracytoplasmic sperm injection, and a twin pregnancy and live birth at 36 weeks followed [77]. That outcome is anecdotal: it arose after drug withdrawal rather than during exposure, it was achieved through assisted reproduction rather than natural conception, and a single uncontrolled observation neither establishes benefit nor offsets the absence of adequately powered fertility trials. One registered phase 2 randomized trial addresses this gap directly but had reported no results at the ClinicalTrials.gov search of 15 August 2026; it is described in Section 11.4 [91]. These reports do not establish a treatment effect on pregnancy or live birth [77,86,89,90].

### 6.4. Clinical Interpretation

The present search identified no eligible controlled tirzepatide-specific semen or fertility study; evidence from classic GLP-1 receptor agonists should not be assumed to apply to the dual GIP/GLP-1 receptor agonist [2]. For men with a metabolic indication, counseling should therefore use measured reproductive findings and the couple’s timeline rather than an expected fertility benefit. Favorable semen signals in metabolically impaired men remain difficult to separate from weight loss [20,68,75].

## 7. Sexual Function and Erectile Dysfunction

The evidence on male sexual function is mixed and design-dependent [76,92,93,94,95]. Prospective studies did not identify a large short-term harm in the populations studied, but they were not designed to establish class-wide sexual safety. In an exploratory analysis of the placebo-controlled REWIND trial—men older than 50 years with type 2 diabetes at high cardiovascular risk, approximately 40% with established cardiovascular disease—dulaglutide was associated with a lower incidence of erectile dysfunction (hazard ratio 0.92, 95% CI 0.85–0.99) [92], and a randomized crossover trial in healthy lean men found no effect of dulaglutide on sexual desire or hypothalamic–pituitary–gonadal hormones [76]. Neither population resembles the man seeking fertility.

Retrospective data point the other way, and the methodologically strongest of them is a target-trial emulation in electronic health records: among men with type 2 diabetes, 4910 initiators of a GLP-1 receptor agonist were compared with 5524 initiators of a dipeptidyl peptidase-4 inhibitor after stabilized inverse-probability-of-treatment weighting, giving erectile-dysfunction incidences of 35.2 versus 28.0 per 1000 person-years and a hazard ratio of 1.26 (95% CI 1.08–1.46); the estimate attenuated and lost statistical significance after calibration against a negative-control outcome, and the pattern was reproduced in an external cohort [93]. Attenuation on negative-control calibration is consistent with residual confounding or other systematic error, so the study is better read as failing to establish harm than as demonstrating it. A database study with no active comparator points the same way on weaker methods: in non-diabetic men with obesity aged 18–50 prescribed semaglutide for weight loss, new erectile dysfunction—a composite of a new diagnosis or a new phosphodiesterase-5 inhibitor prescription—was more frequent (1.47% versus 0.32%; risk ratio 4.5, 95% CI 2.3–9.0), as were testosterone-deficiency diagnoses (risk ratio 1.9, 95% CI 1.2–3.1), on small absolute numbers, with an absolute difference of about 1.15 percentage points, and without ascertainment of fertility intention [94]. Signals of this kind are vulnerable to confounding by indication, differential health-care-seeking, ascertainment bias, the effects of rapid weight loss, and comparator choice [93,94]. Agent-specific sexual-function evidence for the dual GIP/GLP-1 receptor agonist tirzepatide is limited to the controlled initiation pilot, which reported improvement in the five-item International Index of Erectile Function in the tirzepatide arm [2], and a very small late-responder testosterone add-on study [40]. Neither isolates a causal tirzepatide effect, and the latter primarily evaluates testosterone add-on rather than tirzepatide initiation.

Further sources are summarized in Supplementary Table S1 and do not resolve the direction of effect: a narrative review of erectile function; a retrospective cohort of 108 men in whom a long-acting GLP-1 receptor agonist was added to metformin; two analyses of the same spontaneous-reporting database that reach opposite conclusions; a claims-based comparison in which tirzepatide carried a lower risk of incident erectile dysfunction than sitagliptin, injectable semaglutide, or dulaglutide; a narrative review that includes rodent data in which exendin-4 suppressed sexual behavior; a review of sexual dysfunction associated with weight-loss pharmacotherapy; a broader urological review of obesity; and a drug-target Mendelian randomization study reporting a lower risk with genetically proxied exposure [95,96,97,98,99,100,101,102,103]. Current evidence does not establish either a beneficial or harmful class effect on erectile function. New symptoms should be evaluated clinically rather than attributed automatically to GLP-1-based therapy.

## 8. Clinical Positioning Relative to Testosterone Therapy

Many men with obesity or type 2 diabetes who seek fertility care also have low testosterone, and testosterone therapy—the intuitive treatment—is the wrong choice when fertility is desired [20]. Exogenous testosterone suppresses gonadotropin-releasing hormone and, in turn, luteinizing and follicle-stimulating hormone; the resulting fall in intratesticular testosterone impairs spermatogenesis and can cause oligo- or azoospermia. The amended American Urological Association/American Society for Reproductive Medicine (AUA/ASRM) guideline states, as a Clinical Principle—a consensus-based rather than evidence-graded statement—that clinicians should not prescribe exogenous testosterone therapy to men interested in current or future fertility; the Endocrine Society recommends against testosterone therapy in men planning fertility in the near term; and the European Association of Urology (EAU) 2026 guideline states, as strong recommendations, that testosterone therapy is not to be used for the treatment of male infertility or in men wishing to be fathers, and is to be provided in symptomatic primary or secondary hypogonadism only to men who are not considering parenthood [15,16,17,104,105]. Consistent with this, testosterone reduced sperm output in trial data even as it raised serum testosterone [75].

GLP-1-based therapy may be appropriate for an independent metabolic indication in men with obesity-related functional hypogonadism. In a randomized comparison, liraglutide raised gonadotropins relative to transdermal testosterone, although the between-group difference in total testosterone was not statistically significant [80], and, in direct comparison, semaglutide did not reduce semen parameters where testosterone did [75]. A second non-randomized pilot compared continued tirzepatide alone with continued tirzepatide plus testosterone undecanoate in ten late responders [40]. Lean mass declined in the monotherapy group and increased after testosterone add-on, but the very small, selected, non-randomized sample does not establish that tirzepatide causes lean-mass loss. That pilot also shows the gonadotropin trade-off directly: in men aged 35 to 44 years, testosterone undecanoate raised total testosterone from 9.5 ± 1.3 to 18.5 ± 3.4 nmol/L while luteinizing hormone fell from 3.9 ± 1.5 to 0.8 ± 0.3 IU/L and follicle-stimulating hormone from 4.4 ± 1.7 to 1.1 ± 0.4 IU/L, whereas both gonadotropins were unchanged in the men who continued tirzepatide alone [40]. The distinction must not be overstated into general substitution: these agents are licensed as metabolic therapies rather than as treatments for hypogonadism; their testosterone effect is modest and was associated in meta-regression with the degree of weight and body-mass-index loss [18]; they do not correct primary testicular failure [20]; and the present search identified no eligible controlled tirzepatide-specific semen or fertility study [2].

Weight loss and metabolic optimization are foundational and can reverse functional hypogonadism [52,61,106]; when fertility care is time-sensitive, both should proceed in parallel. When symptomatic low testosterone coexists with fertility desire, gonadotropins, human chorionic gonadotropin (hCG), selective estrogen receptor modulators (SERMs), or aromatase inhibitors may be considered according to etiology and under specialist guidance; several of these uses are off-label [15,16,107,108,109,110,111,112,113]. A man may receive GLP-1-based therapy for a valid metabolic indication while gonadotropins support spermatogenesis, provided each is independently justified; the present search identified no eligible study evaluating such co-administration, so this reflects individualized clinical judgment rather than evidence of benefit [15,16].

## 9. Preconception Counseling, Paternal Exposure, and Offspring Health

Human evidence on paternal preconception exposure is insufficient to establish either safety or harm. Pregnancy-related product-label precautions apply to the person who can become pregnant and do not establish an evidence-based washout interval for a male partner. For semaglutide, the product information advises discontinuation at least two months before a planned pregnancy in a patient who can become pregnant, because of the drug’s long half-life; it specifies no paternal interval [114]. Dedicated human data on paternal exposure, semen parameters, and offspring outcomes remain limited: the available studies are small, heterogeneous, and not designed to assess offspring health, and such perinatal data as exist have been reported only in abstract form [115]. The large metabolic trials were powered for glycemic and weight outcomes rather than reproductive endpoints; that absence is neither evidence of safety nor of harm, and no causal paternally mediated adverse reproductive or offspring outcome was established by the sources identified in this review [114,115]. One conference abstract describing a possible preterm-birth signal after paternal exposure is internally inconsistent between its reported confidence interval and its *p*-value, carries residual confounding acknowledged by its authors, and is available only in abstract form, so no effect estimate is quoted here [116]. Evidence on paternal exposure to other antidiabetic agents—meta-analyses of paternal metformin use and a scoping review of paternal preconception health—is indirect: it does not document GLP-1 exposure and cannot substitute for it [117,118,119].

Several concerns are biologically plausible but unproven and should be presented as hypotheses. Paternal metabolic health may influence offspring outcomes, and because these agents alter weight and metabolism they could modify the transmitted paternal phenotype [115,120]; obesity is associated with sperm-epigenome changes that weight loss might alter, in a direction narrative syntheses suggest may be favorable but that has not been demonstrated for GLP-1-based therapy [62,121]; and in type 2 diabetes sperm DNA methylation differs from that of normoglycemic men of similar body mass index, predominantly toward hypermethylation, with stability on repeat sampling at three months and reversibility untested [122]. One human study linked semaglutide to altered DNA methylation of glycolipid-metabolism genes in men with obesity-associated secondary hypogonadism, in an exploratory analysis of very few participants and without sperm tissue, so it establishes nothing about the germline [123]. Animal and in vitro signals neither confirm nor exclude transgenerational effects and cannot be extrapolated to human offspring [62,115].

**Figure 2:**
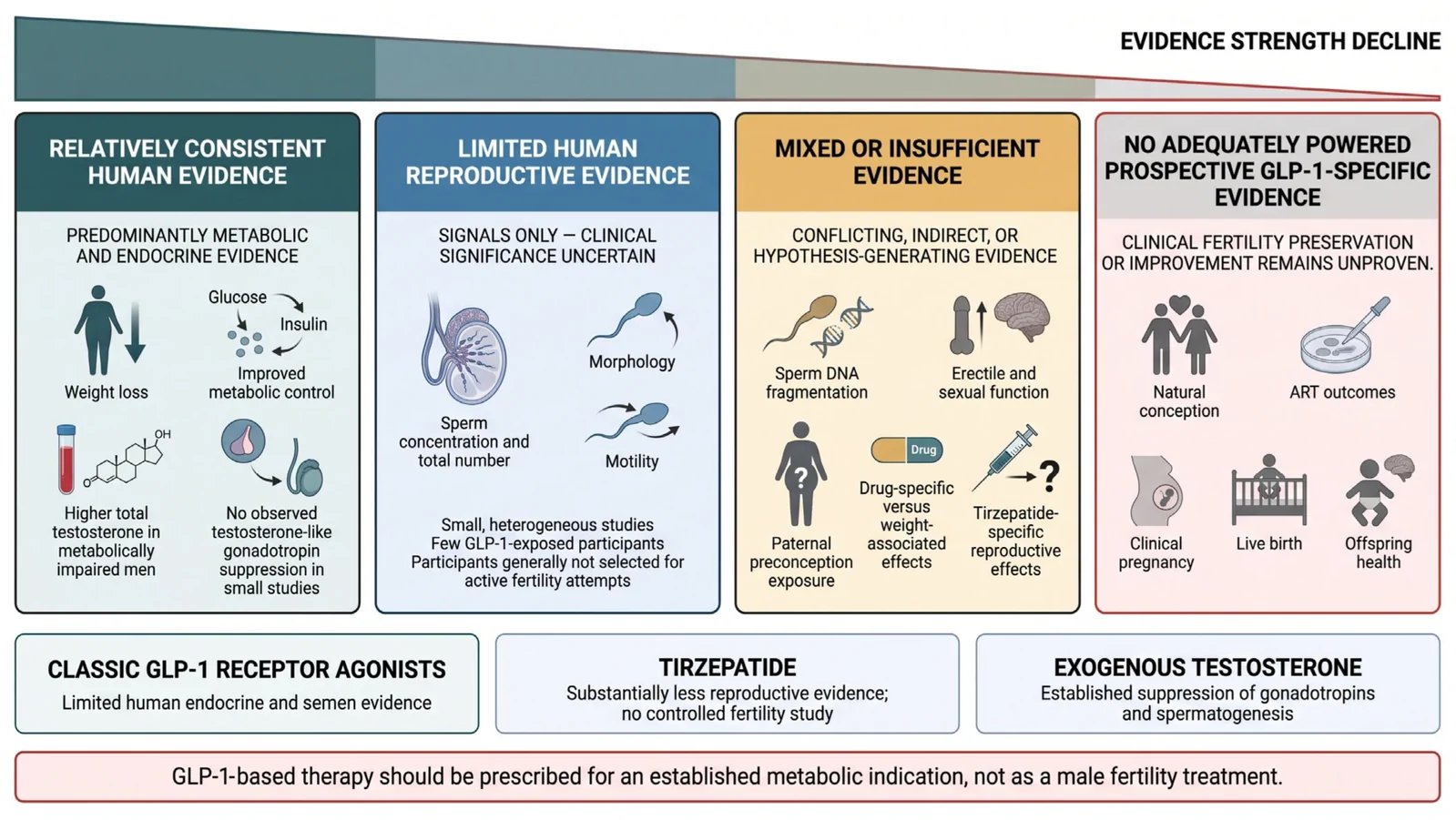
Evidence map across metabolic, endocrine, semen, sexual-function, and paternal-exposure domains. The domains summarize the evidence identified by this review; the lower panels distinguish classic GLP-1 receptor agonists, tirzepatide, and exogenous testosterone. The map describes evidence gaps rather than a formal certainty grading. The tapering bar and the left-to-right order of the columns indicate declining strength of evidence; column colors only separate the four evidence categories. Arrows within icons are illustrative and indicate the direction of a reported change or a link between elements; question marks denote unresolved questions. Abbreviations: ART, assisted reproductive technology; GIP, glucose-dependent insulinotropic polypeptide; GLP-1, glucagon-like peptide-1. Created in BioRender. Kaltsas, A. (2026) https://BioRender.com/keoibkn.

No evidence-based universal male washout interval was identified in the reviewed sources. Continuation, temporary interruption, or switching should be individualized according to the metabolic indication, consequences of treatment withdrawal, tolerability, and the couple’s reproductive timeline. Men seeking fertility should be counseled honestly that these data remain insufficient, that no paternally mediated adverse outcome is established, and that any decision should be shared and made without promising a fertility benefit [62,114,115]. The overall gradient of evidence across metabolic, endocrine, semen, sexual-function, and paternal-exposure domains is summarized in Figure 2.

## 10. A Proposed, Evidence-Informed, Non-Validated Clinical Counseling Framework

**Figure 3:**
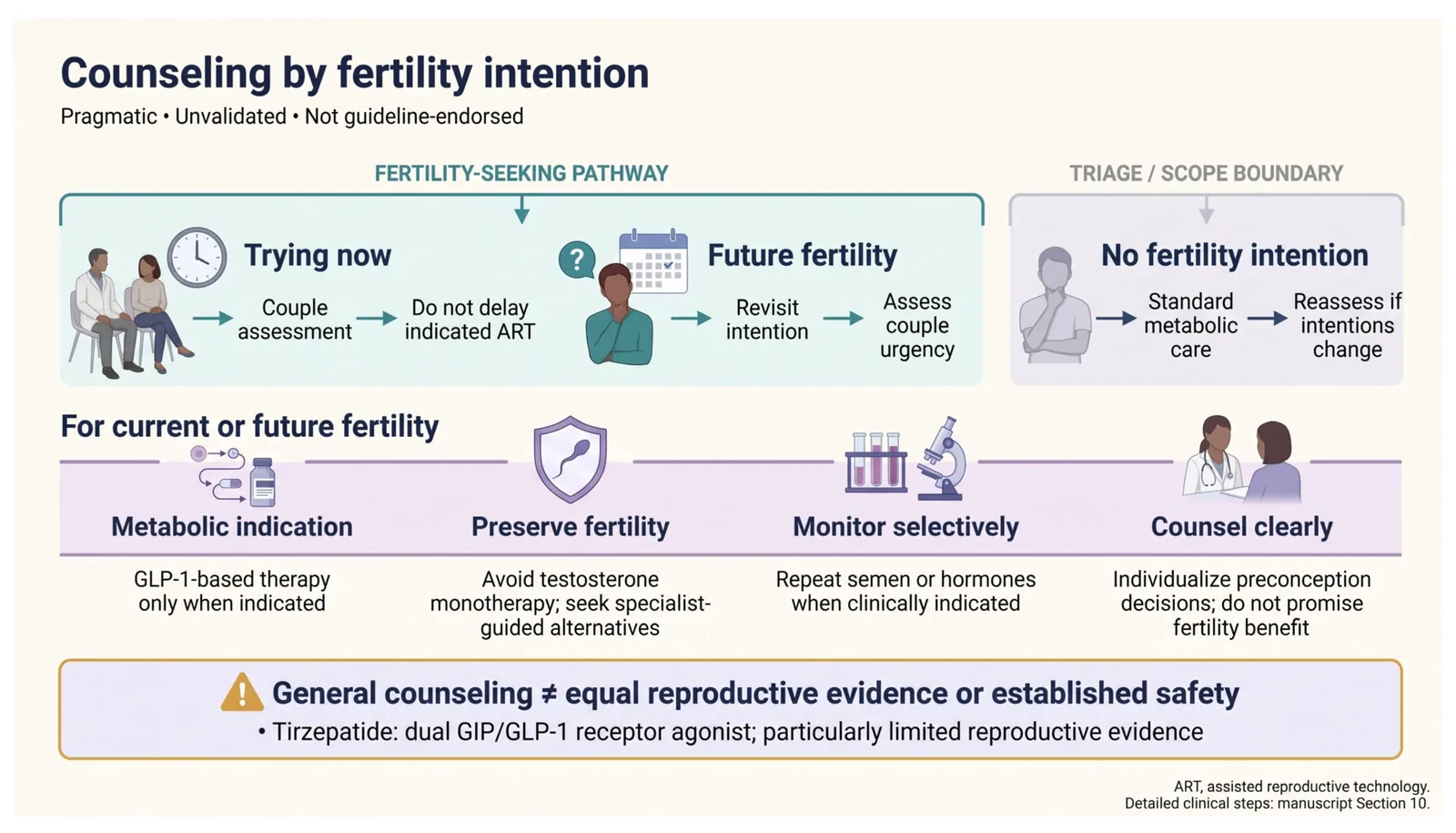
Intention-based counseling framework. The first two categories address current or future fertility; the third marks a triage boundary for men without anticipated fertility intention. General counseling logic does not imply equivalent reproductive evidence or established reproductive safety across agents. The framework is author-developed, evidence-informed, non-validated, and not guideline-endorsed; Section 10 provides provenance tags and detailed clinical steps. Avoidance of testosterone monotherapy follows guideline principles [15,16,17]; the statements on fertility benefit and on tirzepatide-specific evidence rest on the sources reviewed in the text [2,20,40]. Arrows indicate the sequence of counseling steps, and ≠ denotes non-equivalence. Abbreviations: ART, assisted reproductive technology; GIP, glucose-dependent insulinotropic polypeptide; GLP-1, glucagon-like peptide-1. Created in BioRender. Kaltsas, A. (2026) https://BioRender.com/kr88rjg.

The proposed, author-developed, evidence-informed, non-validated framework in Figure 3 supports counseling of men using or considering GLP-1-based therapy who desire current or future fertility. Its third intention category marks the triage boundary outside that target population, as defined in Section 2.1. The framework complements specialist evaluation [124]. Individual statements carry provenance tags: [G] identifies an existing guideline principle and [P] a pragmatic proposal of the present review that has not been prospectively evaluated. These tags do not imply equivalent reproductive evidence or established reproductive safety across agents; tirzepatide-specific evidence is particularly limited [2,20].

Step 1—Define fertility intention. Classify the man as actively attempting conception or undergoing infertility evaluation; as desiring future fertility without an immediate plan; or as having no current or anticipated fertility intention [P]. The third category lies outside the fertility-seeking pathway and is retained to guide triage rather than routine fertility testing. Because intentions change, the question is revisited over time [P]. When current or future fertility is relevant, couple-level urgency is assessed separately from the stated timing of intention and turns on female age, ovarian reserve, duration of infertility, and the presence of a severe male factor [P].

Step 2—Baseline assessment. Assessment is tiered rather than universal: genital examination, semen analysis, and a hormonal panel are not proposed for every man receiving GLP-1-based therapy [P]. Within the fertility-seeking pathway, take a focused reproductive and medication history—prior conceptions and time trying, partner factors and concurrent female-partner assessment, cryptorchidism, testicular surgery or torsion, febrile illness, gonadotoxic exposures, opioids, and explicit inquiry about testosterone and anabolic-androgenic steroid use, a common and reversible cause of impaired spermatogenesis [15,16] [G]. Record body mass index, waist circumference, glycated hemoglobin, and metabolic profile, which establish the legitimacy of the metabolic indication; this metabolic minimum is an author proposal rather than a cited male-fertility guideline recommendation [P]. Perform a genital examination—testicular volume and consistency, epididymes, vasa, and varicocele—within an infertility evaluation or where a clinical indication exists [15,16] [G]. Obtain a semen analysis in men under active infertility evaluation or when the history or examination points to a reproductive abnormality [15,16] [G], or when the result would alter management [P], and interpret it against the sixth-edition reference distribution [27,28] [G]. Obtain a hormonal panel when symptoms suggest hypogonadism, when oligozoospermia or azoospermia is found, when the testes are small or atrophic, or where another specific indication exists; measure morning total testosterone with sex hormone-binding globulin, luteinizing hormone, and follicle-stimulating hormone, adding estradiol and prolactin only where indicated [15,16] [G]. A low morning total testosterone should be confirmed on a second measurement, and free testosterone should be measured by a reliable method—equilibrium dialysis, or calculation from total testosterone, sex hormone-binding globulin, and albumin by a validated method—where sex hormone-binding globulin is altered [17] [G]. A diagnosis of functional hypogonadism requires consistent symptoms, a repeated low morning testosterone, and exclusion of organic causes [17] [G]. Reserve sperm DNA fragmentation testing for specific indications: the AUA/ASRM guideline places karyotype and sperm DNA fragmentation testing within the evaluation of the male partner after failed assisted reproductive technology cycles or two or more pregnancy losses, rather than grading either test itself [15,104] [G]; broader selective indications are discussed in the wider literature [125]; and diabetes is a further reason to consider it selectively, since men with diabetes may carry sperm chromatin damage despite an apparently normal conventional semen analysis [126] [P].

Step 3—Phenotypic stratification. Assign one or more actionable phenotypes: obesity or metabolic dysfunction with functional hypogonadism; eugonadal man with normal semen; abnormal semen parameters; severe male factor; time-sensitive couple; testosterone-therapy candidate; and rapid-weight-loss candidate [P]. The man with obesity and functional hypogonadism is the principal candidate for metabolic therapy that avoids exogenous testosterone, whereas severe male factor or a time-sensitive couple shifts priority toward prompt reproductive-urology assessment with concurrent assisted-reproduction planning [P]. For the man anticipating rapid or large-magnitude weight loss, there is no established reproductive risk; semen parameters have been measured before and after an active dietary weight-loss phase and during pharmacological weight loss without a consistent adverse signal, so no additional testing is proposed on that basis alone [P].

Step 4—Treatment positioning. Avoid testosterone monotherapy whenever fertility is desired [15,16,17] [G]. Use GLP-1-based therapy only for a standard metabolic indication; the reproductive evidence summarized in Section 5 and Section 6 does not establish a fertility indication [20,75,80] [P]. For symptomatic low testosterone with a desire for fertility, consider the alternatives to exogenous testosterone set out in Section 8, under specialist guidance [15,16,17] [G]. Metabolic optimization and fertility care should proceed in parallel rather than sequentially; indicated assisted reproductive technology should not be delayed while weight loss is tried in a time-sensitive couple [P].

Step 5—Monitoring. Metabolic monitoring follows the standard of care for the indication for which the drug was prescribed and lies outside the scope of this review. Serial semen analysis is not proposed solely because GLP-1-based therapy has been initiated [P]. Repeat semen or hormonal testing only when a baseline abnormality, new reproductive or sexual symptoms, active infertility evaluation, or a management decision provides a clinical indication [P]. In men from infertile couples with an abnormal semen analysis, the World Health Organization suggests repeat testing after a minimum of 11 weeks (conditional recommendation; very-low-certainty evidence) [127,128] [G]; azoospermia, severe oligozoospermia, or a time-sensitive couple warrants prompt specialist evaluation without waiting for the repeat interval [P]. New erectile or ejaculatory symptoms should be evaluated for metabolic, vascular, psychosexual, medication-related, and relationship factors rather than attributed to therapy by default [P].

Step 6—Preconception counseling. Explain uncertainty and do not promise improved fertility [P]. Say that sexual function will be assessed clinically and that new symptoms will be investigated, while making clear that current GLP-1-specific evidence is mixed and does not establish erectile dysfunction as a drug effect [94] [P]. Where semen analysis was indicated and repeated, and the intended weight loss has been achieved with parameters unchanged after a further spermatogenic cycle, say so explicitly rather than sustaining an expectation of fertility benefit [P]. Distinguish maternal pregnancy warnings from the paternal data gap without implying teratogenicity [114,115] [P]. Individualize decisions about continuation, pause, or washout to the man’s metabolic needs and the couple’s timeline [P].

Statements marked [P] are anchored to changes that alter clinical management rather than to arbitrary numeric cut-offs, and none has been prospectively evaluated. The framework assumes access to semen analysis, endocrine testing, and specialist services—failing which early referral is preferable—and should be adapted locally and revised as higher-quality data emerge [15,16,27,28]. Its purpose is to structure shared decision-making where evidence is incomplete, not to assert a standard of care.

## 11. Limitations and Future Research

### 11.1. Limitations of the Narrative-Review Method

This is a narrative review, not a systematic review or meta-analysis. The targeted search was confined to a single bibliographic database, PubMed/MEDLINE, supplemented by hand-searching of reference lists, with no registered protocol, duplicate independent screening, PRISMA flow diagram, formal meta-analysis, or study-level risk-of-bias grading, and with attention restricted to English-language sources, so relevant work may have been missed [26]. Statements concerning absent evidence refer to eligible sources identified by this review, retaining the bibliographic cutoff of 10 August 2026. The supplementary search on 10 September 2026 assessed the incremental yield of two added drug terms and was not a full update of the original screening. Registry statements retain the ClinicalTrials.gov search date of 15 August 2026. The included syntheses share overlapping primary studies, so their concordance is not independent replication, and the absence of standardized fertility endpoints prevents quantitative pooling [18,19,20,86].

### 11.2. Limitations of the Evidence Base

The human evidence is heterogeneous in design, agent, dose, population, and endpoint, and mostly concerns metabolic and hormonal rather than fertility outcomes; semen data are sparse and pregnancy and live-birth data essentially absent. Most participants had obesity or type 2 diabetes, which limits generalizability [20,86]. Several key studies are small and open-label, yielding imprecise estimates and possible performance and detection bias [75,80], and follow-up in the four- to eight-week studies is shorter than a single spermatogenic cycle; the 16- to 24-week trials and the 52-week weight-maintenance substudy span at least one cycle but remain short for durability and for hard fertility outcomes [68]. Available designs cannot separate drug effects from weight loss [68]. Paternal preconception exposure and offspring outcomes are insufficiently studied [115], and two analyses of the same pharmacovigilance database reached opposite conclusions [97,99].

### 11.3. Limitations of the Proposed Framework

The proposed framework is author-developed, evidence-informed, and non-validated; it is not guideline-endorsed and was developed as described in Section 2.5. Its provenance tags distinguish guideline-derived principles [G] from pragmatic author proposals [P] but do not grade evidentiary certainty: a [G] statement inherits the certainty and recommendation strength of its source guideline, whereas a [P] statement has not been prospectively evaluated. The reassessment triggers are anchored to management-changing events rather than to arbitrary numeric cut-offs and are offered for testing, not as standards of care.

### 11.4. Future Research Priorities

Closing the gap requires studies designed around male reproduction rather than repurposed from metabolic trials, which were powered for glycemic or weight outcomes and rarely enrolled men by fertility status. Priorities include clinical, mechanistic, and framework-validation studies. First, semen assessment should be standardized against the sixth-edition World Health Organization methods and reference distribution, with transparent reporting [27,28,86]. Second, endocrine endpoints should rest on repeated morning total testosterone with sex hormone-binding globulin and a valid free testosterone measurement, by equilibrium dialysis or validated calculation [17]. Third, weight-matched or pair-fed comparators, or randomization against equivalent non-pharmacological weight loss, are needed to separate direct from weight-mediated effects, building on the weight-maintenance trial [68] and a preclinical pair-fed study [51]. Fourth, agent-specific analyses are required for the dual GIP/GLP-1 receptor agonist tirzepatide and for oral formulations and multi-receptor agonists [2,37,38,39].

Complementary mechanistic studies should test direct effects under controlled in vitro conditions. Building on the reported human-sperm, Sertoli-cell, and organoid observations [11,44,45], paired experiments across multiple human donors are proposed, with vehicle controls, prespecified concentrations and exposure durations, and receptor-specific controls where feasible. Motility, viability, metabolic responses, and DNA integrity should be assessed separately; validated assays of sperm–oocyte interaction or fertilization competence could extend this work. Such experiments would test biological mechanisms under defined conditions and should complement, rather than replace, prospective studies of treated men and couples.

Fifth, mediation analyses should be prespecified rather than reconstructed post hoc, so that the share of any reproductive effect attributable to weight change can be estimated rather than argued. Sixth, adequately powered and ideally randomized studies should prespecify the full causal chain, from semen parameters and sperm DNA fragmentation through natural conception, assisted-reproduction outcomes, clinical pregnancy, and live birth, and should follow couples to conception, with registries capturing the outcomes that trials are unlikely to reach [20,86]. Seventh, registries of paternal preconception exposure and of offspring outcomes are needed, given theoretical concerns around paternal metabolic health and the sperm epigenome and the low-certainty evidence available for paternal antidiabetic exposure [62,115,118,129]. Eighth, the framework proposed here should be validated prospectively against outcomes that matter to couples. Long-term monitoring of sexual function with validated instruments would additionally help resolve the conflicting signals [76,92,93,94,95]. Two prospective registered studies are particularly relevant to the unresolved questions highlighted here. A phase 2 randomized trial compares semaglutide, tirzepatide and a standardized lifestyle intervention in 180 married men aged 20–45 with obesity and diagnosed male infertility, excluding azoospermia, with change in sperm concentration at 32 weeks as its primary endpoint and natural conception within 48 weeks among its secondary endpoints; among the records identified, it was the only registered study that enrolled men by active reproductive intention and carried a non-pharmacological weight-loss comparator, but it was not yet recruiting as of the ClinicalTrials.gov search on 15 August 2026 and had reported no results [91]. A randomized study in men with Klinefelter syndrome carries two co-primary endpoints, sperm retrieval rate at microdissection testicular sperm extraction and change in insulin resistance, but semaglutide sits in the metabolic comparison against testosterone gel rather than in the reproductive one [130]. Among the records identified, no additional registered study carried natural conception, live birth, sperm DNA fragmentation under GLP-1-based therapy, or paternal exposure and offspring outcomes as a primary endpoint.

## 12. Conclusions

GLP-1 receptor agonists and tirzepatide should be used for established metabolic indications rather than as male fertility treatments. In metabolically impaired men, small, heterogeneous studies associate treatment with higher total testosterone, whereas free-testosterone findings are inconsistent and favorable semen signals have not been separated reliably from weight loss. Endocrine improvement does not establish preserved spermatogenesis or improved fertility. The present search identified no eligible, adequately powered prospective GLP-1-specific study assessing sperm DNA fragmentation, conception, assisted-reproduction outcomes, clinical pregnancy, live birth, or offspring health as prespecified endpoints. Tirzepatide requires agent-specific reproductive evidence. Care should integrate metabolic needs, fertility intention, and the couple’s timeline, with avoidance of testosterone monotherapy when fertility is desired. The pragmatic, author-developed, non-validated framework is offered for individualized counseling and prospective evaluation.

## Figures and Tables

**Table 1:** Primary human studies of GLP-1-based therapy with endocrine or semen endpoints, separated by whether a semen endpoint was reported. Panel A comprises the studies reporting semen endpoints; Panel B comprises studies reporting endocrine endpoints only. Denominators are given per arm and per outcome, because the number analyzed differs between outcomes within the same study. Evidence syntheses, sexual-function and pharmacovigilance sources, conference abstracts and paternal-exposure sources are summarized in Supplementary Table S1. Abbreviations: ADAM, Androgen Deficiency in the Aging Male; AMS, Aging Males’ Symptoms; CI, confidence interval; FSH, follicle-stimulating hormone; GIP, glucose-dependent insulinotropic polypeptide; GLP-1, glucagon-like peptide-1; HbA1c, glycated hemoglobin; HOMA, homeostasis model assessment; ICSI, intracytoplasmic sperm injection; IIEF-5, five-item International Index of Erectile Function; LH, luteinizing hormone; SD, standard deviation; SGLT2, sodium–glucose cotransporter-2; SHBG, sex hormone-binding globulin; T2DM, type 2 diabetes mellitus; ΔTT, change in total testosterone.

Study, Design, Comparator and Duration	Population and Fertility Intention	Denominator (Arm- and Outcome-Specific)	Weight and Metabolic Change	Semen or Hormonal Result, with Absolute Values	Within-Group or Between-Group Comparison	Conception or Live Birth Assessed?	Main Limitation
**Panel A. Studies reporting semen endpoints**							
Gregorič 2025 [75]. Randomized, open-label; semaglutide 1 mg/week versus testosterone undecanoate; 24 weeks; NCT06489457	Men with T2DM and functional hypogonadism; fertility intention not an entry criterion; baseline semen quality poor	25 randomized (13 semaglutide, 12 testosterone undecanoate); semen and hormones in both arms	Semaglutide: weight 115 → 99 kg, Δ −6, *p* = 0.004; HbA1c 7.1 → 6.1%, Δ −1.2, *p* = 0.009. Testosterone undecanoate: weight Δ 0.5, *p* = 0.92; HbA1c Δ 0.1, *p* = 0.51. Between-arm *p* = 0.007 and *p* = 0.019. Medians; a median paired change is not necessarily the difference between the two marginal medians. Both arms on a 500–800 kcal/day deficit diet. Waist circumference not reported.	Semaglutide: morphologically normal forms ≈2% → 4% (*p* = 0.012); concentration, total sperm number and motility unchanged; total testosterone rose; LH and FSH essentially unchanged. Testosterone undecanoate: concentration and total sperm number fell	Within-arm for morphology; the between-arm contrast is against an androgen that suppresses spermatogenesis	No	Very small; open-label; no placebo or weight-matched arm; comparator impairs spermatogenesis
Andersen 2022 [68]. Randomized, double-blind, placebo-controlled substudy; 8-week low-calorie diet, then randomization to placebo, exercise, liraglutide 3.0 mg, or liraglutide plus exercise; 52 weeks; NCT04122716	Men with obesity; not enrolled by fertility intention	56 entered; 47 gave the paired diet-phase comparison; 37 analyzed at 52 weeks; 20 exposed to liraglutide	Mean weight loss 16.5 kg (95% CI 15.2–17.8) during the 8-week low-calorie run-in, common to all participants before randomization; 52-week semen gains tracked sustained weight loss irrespective of arm	After the diet: concentration ×1.49 (95% CI 1.18–1.88) and total sperm number ×1.41 (1.07–1.87); motility 33.3% → 31.5%; total motile sperm count 71.3 → 83.7 million/ejaculate (*p* = 0.1); men with <1 million motile sperm 6/47 → 1/47 (*p* < 0.05). At 52 weeks in maintainers, total motile sperm count ×1.50 (0.64–3.52; *p* = 0.3)	Within-group across the diet phase; between-arm at 52 weeks, not powered for reproductive comparison	No	Semen not the primary outcome; not powered for between-arm comparison; attrition 56 → 37
La Vignera 2023 [73]. Prospective comparative, non-randomized; liraglutide 3 mg/day versus gonadotropins versus transdermal testosterone; 4 months	Men with obesity and metabolic hypogonadism of childbearing age; the liraglutide arm comprised men without a current desire for fatherhood	110 across three arms; liraglutide arm 35, all analyzed; no semen data in the testosterone arm	Dietary intervention in all arms (1400–1800 kcal/day). Liraglutide arm: weight 116 ± 10 → 104 ± 6 kg; body mass index 36 ± 3 → 30 ± 2 kg/m^2^; waist 108 ± 6 → 99 ± 4 cm; HOMA index 6.5 ± 1.9 → 3.1 ± 0.6. Mean ± SD; significance reported only as *p* < 0.05. The reported falls in weight (10.3%) and body mass index (16.7%) are not mutually consistent at constant height.	Liraglutide arm: progressive motility 14 ± 2 → 34 ± 4%; total testosterone 1.4 ± 0.6 → 4.1 ± 0.5 ng/mL; SHBG 14.0 ± 3.0 → 36.0 ± 4.0 nmol/L; LH 2.0 → 3.2 and FSH 1.6 → 2.6 IU/L. Concentration, total sperm number, total motility, volume and morphology not reported numerically. Mean ± SD; *p* only as < 0.05.	Within-arm; allocation was by fertility intention, not randomization, so between-arm contrasts are confounded by indication	No	Allocation by fertility intention; short; no pregnancy or assisted-reproduction outcome
Lengsfeld 2024 [76]. Randomized, double-blind, placebo-controlled crossover; dulaglutide; 4 weeks per period; NCT04687514	Healthy lean men; not fertility-seeking	26 randomized; 24 completers, each his own control	Weight change −2.4 kg with dulaglutide versus +0.2 kg with placebo; body mass index −0.7 versus +0.1 kg/m^2^; HbA1c −0.2 versus 0.0%, estimated difference −0.16% (95% CI −0.30 to −0.03). Medians; no *p* values reported.	Only two sperm parameters were reported. Sperm concentration, reported by the source in million sperm per ejaculate rather than per millilitre, 168.8 versus 97.9, estimated difference 36.01 (95% CI −8.64 to 80.65); progressive motility 54.0 versus 56.0%, estimated difference −0.48 (−7.59 to 6.64). Total sperm number, total motility, morphology and volume were measured but not reported. Total testosterone 19.7 versus 19.6 nmol/L, estimated difference 0.92 (−1.47 to 3.32); LH, FSH, SHBG and free testosterone unchanged. Medians; no *p* values.	Within-subject crossover versus placebo	No	Healthy lean men, not generalizable; 4 weeks of exposure is shorter than one spermatogenic cycle, though semen was sampled more than 60 days after the last dose; underpowered; only two sperm parameters reported
Fontoura 2014 [77]. Case report; liraglutide	One man; fertility-seeking	1	Weight 100 kg at initiation, 2 kg lost over five months of exposure. No body mass index, glucose or HbA1c value reported.	No pre-exposure analysis: the first sample was taken one month after liraglutide had started, showing 51.6 million/mL, 55.6% progressive motility, 8% normal forms. At five months of exposure, 0.2 million/mL with no motility, and no spermatozoa on a repeat sample six hours later. After withdrawal: 0.01 million/mL at two months; 8.7 million/mL, 48.1%, 2.5% normal forms at four months; 28 million/mL, 32.1% at five months, morphology not assessed; this sample was used fresh for ICSI. World Health Organization 2010 limits.	Within-subject, uncontrolled	Not an endpoint; twin live birth after ICSI five months after withdrawal	Single case; no true pre-exposure semen analysis; no control; mechanism not established; the live birth followed assisted reproduction after withdrawal, not conception during exposure
**Panel B. Studies reporting endocrine endpoints without any semen endpoint**							
Portillo-Canales 2026 [69]. Retrospective cohort; incretin-based weight-loss drugs, heterogeneous agents and durations; no comparator	Men treated with incretin-based weight-loss drugs; not enrolled by fertility intention	215 with paired total testosterone; 61 with paired free testosterone	Mean weight loss ≈5%	Total testosterone 332 ± 134 → 399 ± 152 ng/dL; free testosterone 6.9 ± 3.0 → 8.0 ± 4.2 ng/dL (*n* = 61); normal testosterone concentration in 67% → 86%	Within-group, paired; no comparator. Testosterone change correlated modestly with weight change (*r* = −0.28 total, −0.26 free)	No	Retrospective; no comparator; heterogeneous agents; paired free testosterone in a minority
Graybill 2021 [78]. Prospective observational cohort; predominantly extended-release exenatide; no comparator; 6 months	Men with type 2 diabetes initiating GLP-1 receptor agonist therapy; not enrolled by fertility intention	51 men with six-month assessment; no semen cohort	Mean weight change −2.27 kg (*p* = 0.0016); HbA1c −0.7 percentage points (*p* = 0.0005)	No significant overall change in total testosterone. In exploratory subgroup comparisons, total testosterone changed from 238.5 ± 56.5 to 272.2 ± 82.3 ng/dL in men with baseline total testosterone below 320 ng/dL, against 438.0 ± 98.2 to 412.0 ± 141.2 ng/dL in men with higher baseline values (between-subgroup *p* = 0.017). A more favorable response was also reported when HbA1c fell by at least one percentage point	Overall within-group analysis; subgroup comparisons were not prespecified	No	Small observational cohort; no comparator; predominantly exenatide; heterogeneous baseline testosterone; concurrent diabetes care; exploratory subgroups; no semen or clinical-fertility outcome
Shao 2018 [79]. Multicenter prospective observational study; exenatide 5 μg twice daily increased to 10 μg twice daily plus metformin 1000 mg twice daily, versus glimepiride 2 mg then 4 mg daily plus metformin 1000 mg twice daily; 12 weeks	Men aged 18 to 70 years with type 2 diabetes, obesity by waist circumference, HbA1c 7.0 to 11.0% and failed glycemic control; fertility intention not an entry criterion	192 enrolled, 176 completed; 90 exenatide and 86 glimepiride	Exenatide arm: weight 88.77 ± 13.55 → 83.04 ± 12.59 kg; body mass index 30.52 ± 3.34 → 28.53 ± 2.89 kg/m^2^; waist 101.63 ± 9.60 → 94.50 ± 9.33 cm; HbA1c 8.38 ± 0.86 → 6.64 ± 0.92%. The glimepiride arm lost 0.75 kg with an equivalent HbA1c fall; between-arm *p* = 0.006 for weight and 0.922 for HbA1c	Total testosterone 378.98 ± 101.42 → 500.70 ± 100.95 ng/dL with exenatide against 380.03 ± 84.68 → 414.70 ± 80.15 with glimepiride; mean rise 121.72 ± 56.73 against 34.67 ± 16.30 ng/dL, between-arm *p* < 0.001. SHBG 18.54 ± 1.70 → 28.90 ± 1.50 nmol/L against 18.53 ± 1.45 → 18.86 ± 1.71, *p* < 0.001. After treatment, neither free testosterone, *p* = 0.884, nor bioavailable testosterone, *p* = 0.840, differed between arms, and LH was unchanged in both, *p* = 0.221. Within the exenatide arm the rise was 142.96 ± 41.55 ng/dL with at least 5% weight loss, 65 men, against 66.48 ± 54.12 with less, 25 men, *p* < 0.001	Between-arm, observational; treatment not allocated by the investigators	No	Observational, not randomized; the comparator sulfonylurea is associated with weight gain, so the between-arm contrast confounds drug with weight trajectory; 12 weeks, limiting assessment of durable reproductive effects; sexual function assessed only by ADAM and AMS questionnaires; no semen, sperm-function or conception outcome
Jensterle 2019 [80]. Prospective, randomized, open-label; liraglutide 3.0 mg versus transdermal testosterone gel; 16 weeks	Men with obesity and functional hypogonadism, poor lifestyle responders	30 randomized (15 liraglutide, 15 testosterone gel); 30 analyzed for hormones; no semen endpoint	Weight change −7.9 ± 3.8 kg with liraglutide versus −0.9 ± 4.5 kg with transdermal testosterone (*p* < 0.001)	Total testosterone +2.6 ± 3.5 nmol/L with liraglutide versus +5.9 ± 7.2 nmol/L with transdermal testosterone; LH and FSH higher with liraglutide	Within-arm rise in both arms; between-arm ΔTT not significant (*p* = 0.239)	No	Small; open-label; endocrine endpoints only
Giagulli 2015 [81]. Retrospective observational; liraglutide added for a second 12 months to lifestyle measures, metformin and testosterone undecanoate in men not reaching the glycemic target; 2 years total	Men with obesity, T2DM, hypogonadism and erectile dysfunction	43 men followed 2 years; liraglutide added in the 26 who did not reach the glycemic target (16 post-pubertal, 10 pre-pubertal onset), while the 17 responders continued without it; no semen endpoint	Liraglutide year only. Post-pubertal subgroup (*n* = 16): weight 99.0 → 93.7 kg, *p* < 0.01; waist 99.1 → 92.5 cm, *p* < 0.001; HbA1c 8.3 → 7.3%, *p* < 0.001. Pre-pubertal subgroup (*n* = 10): weight 102.5 → 98.0 kg, *p* < 0.01; HbA1c 8.2 → 7.4%, *p* < 0.01. Mean ± SD; *p* against the 12-month time point.	Liraglutide year only. Post-pubertal subgroup (*n* = 16): total testosterone 466.1 → 481.7 ng/dL, *p* < 0.001; SHBG 37.1 → 39.1 nmol/L, *p* < 0.01; calculated free testosterone 8.7 → 9.0 ng/dL, not significant. Pre-pubertal subgroup (*n* = 10): total testosterone 395.5 → 420.0 ng/dL, *p* < 0.01; SHBG 38.4 → 40.8 nmol/L, *p* < 0.02. Every man received testosterone undecanoate 1000 mg every 12 weeks throughout. No on-treatment LH or FSH.	Within-group; concurrent exogenous testosterone precludes attribution to liraglutide	No	Retrospective; co-administered testosterone undecanoate in every man precludes attribution; liraglutide given only to the subgroup selected by glycemic non-response; small
Giagulli 2020 [82]. Retrospective single-center study; dulaglutide 1.5 mg weekly in 14 men or liraglutide 1.2 mg daily in 16 men added to metformin, against dapagliflozin 10 mg daily plus metformin in 16 and metformin alone in 25; 12 months	Men aged 45 to 58 years with type 2 diabetes of under 2 years, body mass index at least 30 kg/m^2^, mild to moderate erectile dysfunction and suspected functional hypogonadism; fertility intention not an entry criterion	71 men; 30 exposed to a GLP-1 receptor agonist	Baseline for the whole cohort: weight 104.1 ± 8.3 kg; body mass index 33.7 ± 1.7 kg/m^2^; waist 111.6 ± 5.2 cm; HbA1c 7.7 ± 0.6%	Primary outcome, total testosterone of at least 300 ng/dL at 12 months, reached by 24 of 71, 34%: metformin alone 4%, dapagliflozin 44%, dulaglutide 43%, liraglutide 63%, *p* < 0.01. By weight loss: 3% below 5%, 26% at 5 to 10%, 94% above 10%, *p* < 0.01. By glycemic target: 15% with HbA1c below 6.5% against 51% at 6.5% or above, *p* < 0.01. SHBG rose in every weight-loss stratum, whereas LH did not rise significantly in the men losing most weight. IIEF-5 improved by at least four points in 35 of 71, 49%, and by weight-loss stratum 30% against 65% and 61%, *p* = 0.02	Between-group, retrospective; treatment chosen by prescriber and patient preference	No	Retrospective; not randomized; groups unbalanced at baseline; two GLP-1 receptor agonists and an SGLT2 inhibitor analyzed in parallel; single LH determination without assessment of pulsatility; single study driving the pooled free-testosterone estimate [18]; no semen, sperm-function or conception outcome
La Vignera 2025 [2]. Non-randomized controlled pilot, allocation by patient preference and clinical or economic factors; tirzepatide, a dual GIP/GLP-1 receptor agonist, 2.5 then 5 mg weekly, versus no pharmacotherapy versus transdermal testosterone; 2 months	Men with obesity and metabolic hypogonadism; mean age 55 years; not fertility-seeking	83 enrolled (tirzepatide 28, no pharmacotherapy 30, transdermal testosterone 25); 83 analyzed for endocrine and body-composition endpoints	Diet and exercise advice in all arms. Tirzepatide arm: weight 100.5 → 92.0 kg, *p* < 0.0001; waist 109.0 → 99.0 cm, *p* = 0.0001; HOMA index 5.0 → 4.0, *p* < 0.0001. Between-arm testing was on percentage change: *p* = 0.0007 for weight, *p* < 0.00001 for waist, *p* = 0.01 for HOMA index, the last favoring the testosterone arm. Medians; glucose, insulin and HbA1c not reported.	Tirzepatide arm: total testosterone 186.5 → 424.0 ng/dL, *p* < 0.0001, a median rise of 128.5% against 44.3% with no pharmacotherapy and 46.2% with transdermal testosterone, *p* < 0.00001; LH 2.6 → 4.5 and FSH 1.8 → 3.2 mIU/mL, both of which fell in the testosterone arm; estradiol 33.0 → 11.0 pg/mL; fat mass 17.8 → 10.1% and lean mass 36.2 → 43.5% by bioimpedance. Post-treatment SHBG, free testosterone and IIEF-5 not reported: IIEF-5 appears only as a between-arm percentage change in a figure, without an absolute value or a within-arm *p* value.	Between-arm, but allocation was by patient preference and clinical or economic factors, not randomization	No	Pilot; non-randomized; baseline imbalance in waist circumference, lean mass, LH and binge-eating score, each favoring the tirzepatide arm; 2 months; no semen, DNA-fragmentation or conception outcome
Seminara 2026 [40]. Prospective, non-randomized pilot; continued tirzepatide alone versus continued tirzepatide plus intramuscular testosterone undecanoate 1000 mg; 6 months	Men with obesity and functional secondary hypogonadism classified as late tirzepatide responders after less than 5% weight loss over at least 3 months; age range 35 to 44 years; desire for future paternity was among the factors weighed when allocating to testosterone add-on	10 men, 5 per group	Before enrollment, under tirzepatide monotherapy, lean body mass fell from 66.8 ± 3.5 to 65.2 ± 3.2 kg (*p* < 0.05). At 6 months, lean body mass was 63.4 ± 3.0 kg with tirzepatide alone against 66.1 ± 3.1 kg with testosterone add-on (*p* = 0.009); HOMA-IR 3.8 ± 0.7 against 2.9 ± 0.6 (*p* = 0.009)	Tirzepatide alone, enrollment to 6 months: total testosterone 9.3 ± 1.1 → 10.1 ± 1.3 nmol/L, calculated free testosterone 196.5 ± 22.8 → 212.4 ± 25.1 pmol/L, LH 3.7 ± 1.5 → 4.1 ± 1.5 IU/L, FSH 4.2 ± 1.5 → 4.5 ± 1.7 IU/L and IIEF-5 18.2 ± 1.6 → 18.0 ± 1.5, none significant (*p* ≥ 0.428). Testosterone add-on: total testosterone 9.5 ± 1.3 → 18.5 ± 3.4 nmol/L (*p* = 0.005), LH 3.9 ± 1.5 → 0.8 ± 0.3 and FSH 4.4 ± 1.7 → 1.1 ± 0.4 IU/L (*p* < 0.01), IIEF-5 18.0 ± 1.8 → 23.2 ± 2.1 (*p* = 0.007)	Between-group, non-randomized comparison; neither arm represents new tirzepatide initiation	No	Extremely small and selected sample, 5 per arm; non-randomized, allocation by counseling and preference; all participants had prior tirzepatide exposure; exogenous testosterone prevents attribution of endocrine effects to tirzepatide; no semen or clinical-fertility outcome

## Data Availability

No new data were created or analyzed in this study. Data sharing is not applicable to this article.

## Supplementary Materials

The following supporting information can be downloaded at: https://www.mdpi.com/article/10.3390/jcm15187313/s1, Table S1, Evidence syntheses, sexual-function and pharmacovigilance sources, conference abstracts, and paternal-exposure sources; Table S2, Full search strategy, audit details, and inventory of reports by evidence area.

## Abbreviations

The following abbreviations are used in this manuscript:

ADAM	Androgen Deficiency in the Aging Male
AMS	Aging Males’ Symptoms
ART	assisted reproductive technology
ASRM	American Society for Reproductive Medicine
AUA	American Urological Association
CI	confidence interval
EAU	European Association of Urology
FSH	follicle-stimulating hormone
GIP	glucose-dependent insulinotropic polypeptide
GLP-1	glucagon-like peptide-1
GRADE	Grading of Recommendations Assessment, Development and Evaluation
HbA1c	glycated hemoglobin
hCG	human chorionic gonadotropin
HOMA	homeostasis model assessment
ICSI	intracytoplasmic sperm injection
IIEF-5	five-item International Index of Erectile Function
LH	luteinizing hormone
PRISMA	Preferred Reporting Items for Systematic Reviews and Meta-Analyses
ROBINS-I	Risk Of Bias In Non-randomized Studies of Interventions
SANRA	Scale for the Assessment of Narrative Review Articles
SD	standard deviation
SERM	selective estrogen receptor modulator
SGLT2	sodium–glucose cotransporter-2
SHBG	sex hormone-binding globulin
SHIM	Sexual Health Inventory for Men
STROBE	Strengthening the Reporting of Observational Studies in Epidemiology
T2DM	type 2 diabetes mellitus
