# Supplementary material for: GLP-1 Receptor Agonists and Tirzepatide in Men Seeking Fertility: A Structured Narrative Review and Proposed Clinical Framework
Source: J Clin Med. 2026 Sep 20;15(18):7313. doi: 10.3390/jcm15187313 (PMC13607358; doi:10.3390/jcm15187313)
Supplement: Supplementary file 1 [file jcm-15-07313-s001.zip › 04_Supplementary_Table_S1.pdf]

Supplementary Material

GLP-1 Receptor Agonists and Tirzepatide in Men Seeking Fertility: A Structured Narrative Review and Proposed Clinical Framework

Table S1. Evidence syntheses, sexual-function and pharmacovigilance sources, conference abstracts, and paternal-exposure sources. Table 1 of the main text presents the primary studies selected for the core endocrine and semen synthesis; the present table summarizes complementary evidence syntheses, sexual-function and pharmacovigilance studies, abstract-only primary reports, and paternal-exposure sources. Reference numbers correspond to the main reference list.

Source	Design	Population	Size	Outcomes reported	Main limitations
**Evidence syntheses**					
Deameh 2026 [20]	Systematic review	Adult men (obesity, T2DM, functional hypogonadism, healthy)	10 studies; 639 men	↑ total testosterone, especially in metabolically impaired men; free testosterone inconsistent (offset by ↑ SHBG); LH/FSH preserved or increased; semen improved in obese or hypogonadal but not healthy men	Appraised at abstract level; heterogeneous RCT, crossover and cohort designs; no standardized fertility endpoints; no conception, pregnancy or live-birth outcome
Salvio 2025 [18]	Systematic review and meta-analysis	Men with testicular dysfunction	7 studies; 680 men	Standardized mean difference for total testosterone 1.39 (95% CI 0.70–2.09), 0.96 (0.48–1.44) after removing the principal source of heterogeneity; meta-regression correlated with weight loss	Heterogeneity; overlaps other syntheses; before–after cohorts
Corona 2026 [19]	Systematic review and meta-analysis	Men with metabolic disease	8 GLP-1 RA studies, 375 men; 3 SGLT2i studies, 52 men	Sexual hormones and behaviors; testosterone effect reported as independent of weight loss, interpreted by the authors as a direct mechanism	Online ahead of print; combines GLP-1 RAs with SGLT2 inhibitors; aggregated heterogeneous data
Peel 2026 [86]	Systematic review and meta-analysis	Men undergoing obesity interventions	32 studies (pharmacotherapy in 5)	Pooled estimate for liraglutide null: mean difference 0.58 million/mL (95% CI −24.76 to 25.91), P = 0.96; improvements attributed to lifestyle rather than pharmacological weight loss	GRADE very low certainty; indirect (obesity interventions broadly); conception and ART data limited
Orra 2025 [74]	Systematic review and meta-analysis	Men receiving GLP-1 receptor agonists	4 studies; 219 men	Significant change in bioavailable testosterone and fall in HbA1c; free testosterone and SHBG unchanged	Direction of the pooled estimate reported inconsistently in the abstract; small; I^2 = 86%

Source	Design	Population	Size	Outcomes reported	Main limitations
Santi 2025 [88]	Meta-analysis	Men with obesity undergoing weight loss	12 studies; 345 men	DNA fragmentation index reduced (effect size −0.689, 95% CI −1.123 to −0.255; p = 0.002); concentration and progressive motility increased	Not GLP-1-specific; heterogeneous interventions
Zafrani 2026 [83]	Systematic review	Adult men with obesity, T2DM, functional hypogonadism, or healthy eugonadal controls	Eight studies in the flow diagram and results table; the printed study-level sample sizes sum to 466	Phenotype-dependent endocrine signal; no significant endocrine or reproductive effect in the one placebo-controlled crossover trial in healthy eugonadal men; certainty rated by the authors as low for total testosterone, low to very low for free testosterone and SHBG, very low for semen parameters and moderate for metabolic outcomes; no conception or live-birth outcome	Narrative synthesis only, heterogeneity precluded pooling; search restricted to January 2021 to January 2026, so several eligible primary studies are absent; 28 of 51 full texts could not be retrieved; the text reports seven included studies against eight in the flow diagram, abstract and results table, and approximately 1200 participants against 466 when the printed sample sizes are summed; Gregorič is classified as a randomized trial in the results table yet assessed with ROBINS-I in the non-randomized risk-of-bias table, which lists seven studies although the text says six; the Highlights present the class as a fertility-preserving strategy although the review contains no conception or live-birth data
Olivares 2026 [84]	Narrative review	Men with obesity or T2DM; broad male sexual, endocrine, and reproductive scope	Not applicable	Metabolic mechanisms, hypogonadism, erectile dysfunction, semen parameters, and male fertility	Narrative and mechanistically broad; no quantitative synthesis and no new primary clinical-fertility outcome; substantial overlap with existing reviews; statements suggesting restoration of fertility, superiority to gonadotropins or testosterone, or prevention of unintended pregnancy exceed the underlying evidence
**Sexual-function studies, pharmacovigilance analyses, and mechanistic sources**					
Bajaj 2021, REWIND [92]; NCT01394952	Exploratory analysis of a randomized trial	Men >50 years with T2DM at high cardiovascular risk (~40% established disease)	3725 men	Lower erectile-dysfunction incidence with dulaglutide (hazard ratio 0.92, 95% CI 0.85–0.99)	Exploratory; questionnaire-based; population unlike men seeking fertility

Source	Design	Population	Size	Outcomes reported	Main limitations
Tang 2026 [93]	Target trial emulation (electronic health records)	Men with T2DM, single health system	4910 vs 5524 after IPTW weighting	↑ erectile dysfunction (hazard ratio 1.26, 95% CI 1.08–1.46); not significant after negative-control-outcome calibration	Observational; residual confounding
Able 2025 [94]	Retrospective database (TriNetX)	Non-diabetic men with obesity, 18–50 years	3094 matched pairs	Composite new erectile-dysfunction diagnosis or PDE5-inhibitor prescription 1.47% vs 0.32% (risk ratio 4.5, 95% CI 2.3–9.0); testosterone-deficiency diagnosis risk ratio 1.9 (1.2–3.1)	Absolute difference ~1.15 percentage points; no active comparator; no fertility-intention ascertainment; ascertainment bias
Lisco 2024 [96]	Retrospective cohort, 12 months; long-acting GLP-1 receptor agonist added to metformin	Men with T2DM complaining of erectile dysfunction	108 men	Weight loss, better glycemic control, higher testosterone and improved IIEF-5 scores at 12 months	Retrospective; association only; concurrent metabolic improvement confounds attribution to the drug
Cowart 2025 [98]	Retrospective claims analysis	Men 18–70 years with T2DM, no prior erectile dysfunction	Three 1:1 propensity-matched comparisons	Lower risk with tirzepatide vs sitagliptin (0.70, 0.64–0.76), injectable semaglutide (0.67, 0.62–0.72) and dulaglutide (0.55, 0.51–0.59)	Claims-based; same database family as [93]; residual confounding
Pourabhari Langroudi 2025 [97]; Lin 2026 [99]	Pharmacovigilance disproportionality (FAERS)	Spontaneous adverse-event reports	182 reports; separate class-wide analysis	Opposite conclusions from the same database: low, non-significant disproportionality in one; significant semaglutide signal (reporting odds ratio 1.53, 95% CI 1.16–2.03) in the other	Spontaneous reporting; no denominator; no causal inference
Kounatidis 2025 [95]	Narrative review	Men receiving GLP-1 receptor agonists	Not applicable	Erectile function; the direction of effect is presented as unsettled	Narrative; no quantitative synthesis; draws on the same primary sources listed above
Merhi 2026 [100]	Narrative review, including preclinical data	Women and men	Not applicable	Emerging sexual-function evidence; exendin-4 suppressed sexual behavior in rodents	Narrative; rodent data not translatable to men; sexual-function endpoints have been secondary in existing trials
Fuentes-Mendoza 2026 [101]	Narrative review	Users of weight-loss pharmacotherapy	Not applicable	Sexual dysfunction as an under-reported outcome of weight-loss drugs	Narrative; no quantitative synthesis; not GLP-1-specific

Source	Design	Population	Size	Outcomes reported	Main limitations
Khalil 2026 [102]	Systematic review	Adults with morbid obesity treated by bariatric surgery or GLP-1 receptor agonists; male reproductive outcomes are one subdomain of a broader urological review	84 studies; more than 165,000 participants	Improvements in testosterone and erectile function reported	No semen or clinical-fertility endpoints; indirect, covering obesity interventions rather than GLP-1-specific effects
An 2024 [103]	Drug-target Mendelian randomization	Genetically proxied GLP-1 receptor agonist exposure	22 cis-eQTL variants for GLP1R expression, from 31,684 blood samples; erectile-dysfunction outcome 6175 cases and 217,630 controls; F statistics 47.19–52.93	Lower risk of erectile dysfunction (odds ratio 0.49, 95% CI 0.43–0.57)	A genetic proxy is not drug exposure; the authors call for confirmation in randomized trials
**Conference abstracts and paternal-exposure sources**					
Eid 2025 [85]	Conference abstract; retrospective chart review	Men prescribed GLP-1 receptor agonists for obesity or T2DM	53 charts reviewed; 14 men with paired total testosterone and 5 with paired SHIM scores	Total testosterone 247 → 358 ng/dL, mean increase 111 ng/dL ($t = -2.19$, $p = 0.048$) in 14 men; SHIM 12.4 → 14.8, mean increase 2.4 points, not significant (Wilcoxon $W = 10.5$, $p = 0.42$) in 5 men; no association between weight change and testosterone change ($R^2 = 0.000$, $p = 0.969$); mean weight change −6.3% and HbA1c change −0.8%	Abstract only; the analyzed denominators are 14 and 5 against a 53-chart cohort; individual agents and treatment durations not specified; no comparator; wide ranges (testosterone change −83 to +611 ng/dL; SHIM change −4 to +10); no semen, conception, pregnancy, or live-birth outcome

Source	Design	Population	Size	Outcomes reported	Main limitations
Hviid 2026, ESHRE [89]	Conference abstract; nationwide registry cohort with intention-to-treat emulation and inverse probability of treatment weighting (Denmark, 2018–2024)	Couples undergoing intrauterine insemination or IVF/ICSI; exposure defined as redeemed semaglutide prescription	38,399 women and their male partners; 133 men exposed before IUI, 183 before IVF/ICSI	Male exposure: clinical pregnancy after IUI odds ratio 1.52 (95% CI 1.10–2.09); first IVF/ICSI cycle 0.75 (95% CI 0.58–0.96); no consistent association after female exposure	Conference abstract, not fully peer reviewed; estimates point in opposite directions across modalities; exposure is semaglutide only despite the class wording; no adjustment for weight change or regain; timing relative to conception not quantified
Ahsan 2026, SfE BES [90]	Conference abstract; single case report	Man aged 42 years with obesity, T2DM, erectile dysfunction and azoospermia	n = 1	Substantial weight loss over 8 months on sequential semaglutide then tirzepatide; total testosterone 3.2 → 7.4 nmol/L; inhibin B 111.5 pg/mL; concentration 0 → 31 million/mL, total sperm number 0 → 164.3 million, motility 0 → 39%; natural conception	Anecdotal; sequential exposure to two agents cannot separate drug from weight loss; single azoospermic baseline not reported as confirmed on a second sample; the reported weight and BMI values are internally inconsistent (123 kg at BMI 40.6 implies 1.74 m, so a stated 16.3 kg loss gives BMI 35.2, not 32.1) and are not used quantitatively; post-treatment morphology remained below the reference threshold
Sellke 2026 [116]	Conference abstract; database analysis (MarketScan 2007–2023)	Fathers with preconception GLP-1 receptor agonist exposure	639 men per matched cohort	As printed: preterm birth 9.7% vs 5.6% and NICU admission 15.6% vs 12.1%, both $p = 0.06$; odds ratio for preterm birth 1.80 (95% CI 1.17–2.76) for exposure within 90 days. These are not mutually compatible and no effect estimate is carried into the main text	Conference abstract only; the 95% CI excludes 1 while the stated $p = 0.06$ does not, and at 639 per group the quoted percentages imply $p \approx 0.006$ for preterm birth, so the estimate is internally inconsistent and unusable; confounding by indication acknowledged by the authors; concerns preterm birth and neonatal care, not congenital malformation
Damkier 2026 [117]; Simonsen 2025 [118]	Systematic reviews and meta-analyses	Paternal antidiabetic and metformin exposure	4 population-based datasets each; Damkier 11,508 exposed men	Damkier: pooled adjusted risk ratio 1.03 (95% CI 0.88–1.21), $I^2 = 64\%$; 0.93 (0.83–1.04) with $I^2 = 0\%$ when the Danish data are excluded. Simonsen: pooled adjusted odds ratio 1.05 (95% CI 0.95–1.17), $I^2 = 76.9\%$	Indirect (metformin, not GLP-1 receptor agonists); not independent — Damkier notes that Simonsen pooled two Danish datasets overlapping by ≥90% and omitted the US cohort; low certainty

Abbreviations: ART, assisted reproductive technology; CI, confidence interval; FAERS, FDA Adverse Event Reporting System; FSH, follicle-stimulating hormone; GLP-1 RA, glucagon-like peptide-1 receptor agonist; GRADE, Grading of Recommendations Assessment, Development and Evaluation; HbA1c, glycated hemoglobin; IPTW, inverse probability of treatment weighting; LH, luteinizing hormone; NICU, neonatal intensive care unit; PDE5, phosphodiesterase-5; SGLT2i, sodium–glucose cotransporter-2 inhibitor; SHBG, sex hormone–binding globulin; SHIM, Sexual Health Inventory for Men; T2DM, type 2 diabetes mellitus.
