# Supplementary material for: GLP-1 Receptor Agonists and Tirzepatide in Men Seeking Fertility: A Structured Narrative Review and Proposed Clinical Framework
Source: J Clin Med. 2026 Sep 20;15(18):7313. doi: 10.3390/jcm15187313 (PMC13607358; doi:10.3390/jcm15187313)
Supplement: Supplementary file 1 [file jcm-15-07313-s001.zip › 05_Supplementary_Table_S2.pdf]

Supplementary Material

GLP-1 Receptor Agonists and Tirzepatide in Men Seeking Fertility: A Structured Narrative Review and Proposed Clinical Framework

Table S2. Full search strategy and audit details for each information source. PubMed/MEDLINE was the only bibliographic database searched. The bibliographic evidence cutoff was 10 August 2026; ClinicalTrials.gov was searched on 15 August 2026. The review is a narrative synthesis; no registered protocol, duplicate independent screening, or PRISMA flow diagram was undertaken.

Source and platform	Date run	Complete query as executed	Limits applied	Records retrieved
PubMed/MEDLINE (NCBI)	10 August 2026; identical query rerun for audit on 15 August 2026	("glucagon-like peptide 1"[tiab] OR "GLP-1"[tiab] OR semaglutide[tiab] OR liraglutide[tiab] OR dulaglutide[tiab] OR exenatide[tiab] OR tirzepatide[tiab] OR orforglipron[tiab] OR retatrutide[tiab]) AND (male[tiab] OR men[tiab] OR paternal[tiab] OR sperm*[tiab] OR semen[tiab] OR testosterone[tiab] OR hypogonadism[tiab] OR "erectile dysfunction"[tiab] OR fertilit*[tiab] OR preconception[tiab])	None applied at search; English-language restriction applied during source selection	Original contemporaneous yield not retained; independent audit rerun on 15 August 2026: 3,141 records
ClinicalTrials.gov	15 August 2026	Intervention/treatment: ("glucagon-like peptide-1 receptor agonist" OR "GLP-1 receptor agonist" OR semaglutide OR liraglutide OR dulaglutide OR exenatide OR tirzepatide OR orforglipron OR retatrutide); Outcome measure: (sperm OR semen OR testosterone OR hypogonadism OR "erectile dysfunction" OR "erectile function" OR fertility OR infertility OR reproductive OR preconception OR paternal OR pregnancy OR "live birth"); Other terms: (male OR men OR paternal)	No recruitment-status, study-type, phase, country, age, date, or language filters; records screened for relevance to adult male reproductive health	13
Regulatory and guideline sources	20 June to 10 August 2026 (access dates recorded in the reference list)	Retrieved directly from the issuing bodies (US Food and Drug Administration product labeling and safety communications; WHO; EAU; AUA/ASRM)	Not a database search	Not applicable
Hand-searching	Iteratively through 10 August 2026	Reference lists of relevant reviews [1,5,20]	Not a database search	Not separately recorded; reference-list checking was not maintained as a distinct record set

Audit note: The historical PubMed count from 10 August 2026 could not be recovered from a contemporaneous search history or export. The unchanged query was therefore rerun on 15 August 2026 and retrieved 3,141 records. The rerun documented the current yield and identified late-indexed material; it did not redefine the bibliographic eligibility cutoff of 10 August 2026. Directly relevant audit-identified reviews were appraised contextually, and citation chasing identified the pre-cutoff Graybill 2021 prospective cohort and the Eid 2025 conference abstract, both of which were incorporated into the review. Reference-list checking was iterative and no separate hand-search log was retained. The ClinicalTrials.gov search used API v2.0.5, whose data snapshot (timestamp 2026-08-14T09:00:05) preceded the query by one day, and retrieved NCT02527200, NCT02918279, NCT03619330, NCT04122716, NCT04687514, NCT04881110, NCT05586802, NCT05598008, NCT06489457, NCT06565611, NCT07169136, NCT07179120, and NCT07307235.

Supplementary search during revision

On 10 September 2026, lixisenatide and albiglutide were added to the intervention terms. The original and expanded queries were executed with identical publication-date limits through 10 August 2026, preserving the original eligibility cutoff. No other search filters were applied; the English-language restriction was applied during selection. The queries retrieved 3,150 and 3,154 records. Their difference comprised four records, all assessed for relevance; none was eligible for the male reproductive evidence synthesis.

These are current rerun counts. The historical audit count of 3,141 on 15 August 2026 is retained above. The original contemporaneous yield remains unavailable. Unlike the historical query, which applied the cutoff during eligibility assessment, the current matched queries apply an explicit publication-date limit. Indexing changes and this difference prevent interpreting the counts as simple record accrual. This exercise assessed the added terms and was not a full rescreening or an extension of the review cutoff.

Original terms with matched date limit

("glucagon-like peptide 1"[tiab] OR "GLP-1"[tiab] OR semaglutide[tiab] OR liraglutide[tiab] OR dulaglutide[tiab] OR exenatide[tiab] OR tirzepatide[tiab] OR orforglipron[tiab] OR retatrutide[tiab]) AND (male[tiab] OR men[tiab] OR paternal[tiab] OR sperm*[tiab] OR semen[tiab] OR testosterone[tiab] OR hypogonadism[tiab] OR "erectile dysfunction"[tiab] OR fertilit*[tiab] OR preconception[tiab]) AND ("1000/01/01"[Date - Publication] : "2026/08/10"[Date - Publication])

Expanded terms with matched date limit

("glucagon-like peptide 1"[tiab] OR "GLP-1"[tiab] OR semaglutide[tiab] OR liraglutide[tiab] OR dulaglutide[tiab] OR exenatide[tiab] OR tirzepatide[tiab] OR orforglipron[tiab] OR retatrutide[tiab] OR lixisenatide[tiab] OR albiglutide[tiab]) AND (male[tiab] OR men[tiab] OR paternal[tiab] OR sperm*[tiab] OR semen[tiab] OR testosterone[tiab] OR hypogonadism[tiab] OR "erectile dysfunction"[tiab] OR fertilit*[tiab] OR preconception[tiab]) AND ("1000/01/01"[Date - Publication] : "2026/08/10"[Date - Publication])

Additional record screening

PMID 38877681. Lixisenatide with or without ticagrelor in male rats. Excluded: aortic vascular and metabolic endpoints; no eligible male reproductive endpoint described. Title and abstract assessed.

PMID 36376644. ENSURE retrospective study of insulin glargine and lixisenatide. Excluded: glycemic control, weight, treatment use, and hypoglycemia; no eligible male reproductive endpoint reported. Title, abstract, and full text assessed.

PMID 30410285. 2018 European Association for the Study of Diabetes meeting report. Excluded: albiglutide cardiovascular findings and testosterone therapy appeared in separate sessions; no reproductive effect of albiglutide described. Title and abstract assessed.

PMID 27516879. DPP-4 inhibitor review in chronic kidney disease. Excluded: glycemic outcomes with an albiglutide comparator; no eligible male reproductive endpoint reported. Title, abstract, and full text assessed.

The complete PMID sets and raw PubMed E-utilities exports were retained in the revision audit. The additional-only query and the set difference between the matched queries returned identical records. No new reference was added to the manuscript on the basis of this supplementary search.

Inventory of reports by evidence area

Counts refer to distinct cited reports, not independent datasets, participants, or a reconstructed study-selection flow. A report can contribute to more than one domain. Reviews can include overlapping primary studies; domain counts must not be summed. Reference numbers below follow the manuscript.

Core human evidence: Table 1 includes 13 reports. Five report semen endpoints [68,73,75–77]; eight report endocrine endpoints without semen assessment [2,40,69,78–82]. Eleven of the 13 include measured hormone outcomes [2,40,69,73,75,76,78–82]. The conference case [90] provides additional semen and hormone observations outside the core table.

Supplementary evidence: Table S1 includes 26 reports, consisting of 14 syntheses or reviews [18–20,74,83,84,86,88,95,100–102,117,118], five additional clinical sexual-function reports [92–94,96,98], two pharmacovigilance reports [97,99], one genetic-proxy analysis [103], and four conference abstracts [85,89,90,116].

Sexual function: eight published clinical reports are discussed in Section 7 [2,40,76,92–94,96,98]. Additional sexual outcomes appear in four other core-table reports [73,80–82] and the conference abstract [85]. Pharmacovigilance [97,99] and genetic-proxy findings [103] are counted separately from clinical treatment reports.

Clinical fertility and paternal exposure: three reports describe conception, pregnancy, or live birth [77,89,90], comprising one published case report and two conference reports. Two conference reports directly assess paternal exposure in relation to pregnancy or perinatal outcomes [89,116]; [89] overlaps the clinical-fertility group. These counts do not imply efficacy or causal exposure effects.

Mechanistic evidence: 14 experimental-model reports [11,12,41–49,51,72,87] are distinguished from two human native-GLP-1 infusion reports [13,50]. The latter overlap the endocrine discussion. Systemic methylation [123] and diabetes-related sperm methylation [122] are separately labeled contextual observations. Two prospective registrations without results [91,130] are considered separately from completed reports.
